# Isolation and Characterization of a Novel Rebaudioside M Isomer from a Bioconversion Reaction of Rebaudioside A and NMR Comparison Studies of Rebaudioside M Isolated from *Stevia rebaudiana* Bertoni and *Stevia rebaudiana* Morita

**DOI:** 10.3390/biom4020374

**Published:** 2014-03-31

**Authors:** Indra Prakash, Cynthia Bunders, Krishna P. Devkota, Romila D. Charan, Catherine Ramirez, Christopher Priedemann, Avetik Markosyan

**Affiliations:** 1 The Coca-Cola Company, Atlanta, GA 30313, USA; E-Mail: cbunders@coca-cola.com; 2 AMRI-Albany, Analytical Development, Albany, NY 12212, USA; E-Mails: Krishna.Devkota@amriglobal.com (K.P.D.); Romila.Charan@amriglobal.com (R.D.C.); Catherine.Ramirez@amriglobal.com (C.R.); Christopher.Priedemann@amriglobal.com (C.P.); 3 PureCircle Limited, Lengkuk Teknologi, Bandar Enstek, Negeri Sembilan 71760, Malaysia; E-Mail: avetik@purecircle.com

**Keywords:** *Stevia rebaudiana* Bertoni, *Stevia rebaudiana* Morita, rebaudioside M, rebaudioside D, rebaudioside M2, bioconversion, steviol glycosides, structure characterization, nuclear magnetic resonance (NMR)

## Abstract

A minor product, rebaudioside M2 (**2**), from the bioconversion reaction of rebaudioside A (**4**) to rebaudioside D (**3**), was isolated and the complete structure of the novel steviol glycoside was determined. Rebaudioside M2 (**2**) is considered an isomer of rebaudioside M (**1**) and contains a relatively rare 1**→**6 sugar linkage. It was isolated and characterized with NMR (^1^H, ^13^C, COSY, HSQC-DEPT, HMBC, 1D-TOCSY, and NOESY) and mass spectral data. Additionally, we emphasize the importance of 1D and 2D NMR techniques when identifying complex steviol glycosides. Numerous NMR spectroscopy studies of rebaudioside M (**1**), rebaudioside D (**3**), and mixture of **1** and **3** led to the discovery that SG17 which was previously reported in literature, is a mixture of rebaudioside D (**3**), rebaudioside M (**1**), and possibly other related steviol glycosides.

## 1. Introduction

Sweetness is universally regarded as pleasant and preferred taste for beverages, food, pharmaceuticals, and oral hygiene/cosmetic products. To provide sweet taste to consumer products the most commonly used natural caloric sugars are sucrose, fructose, and glucose. Since these natural sugars provide calories, alternative sources must be utilized when the consumer desires a sweet taste with low to no calories. Artificial and natural sweeteners have been developed to fulfill both criteria [1,2]. It is not a simple task to create a non-caloric or low calorie sweetener because they exhibit a temporal profile, maximal response, flavor profile, mouth feel, and/or adaptation behavior that differ from sugar [3,4]. Steviol glycosides isolated from *Stevia rebaudiana* Bertoni have been explored to produce an ideal sweetener (sweet, low to no calorie, and natural) [5,6,7,8,9,10,11,12].

**Figure 1:**
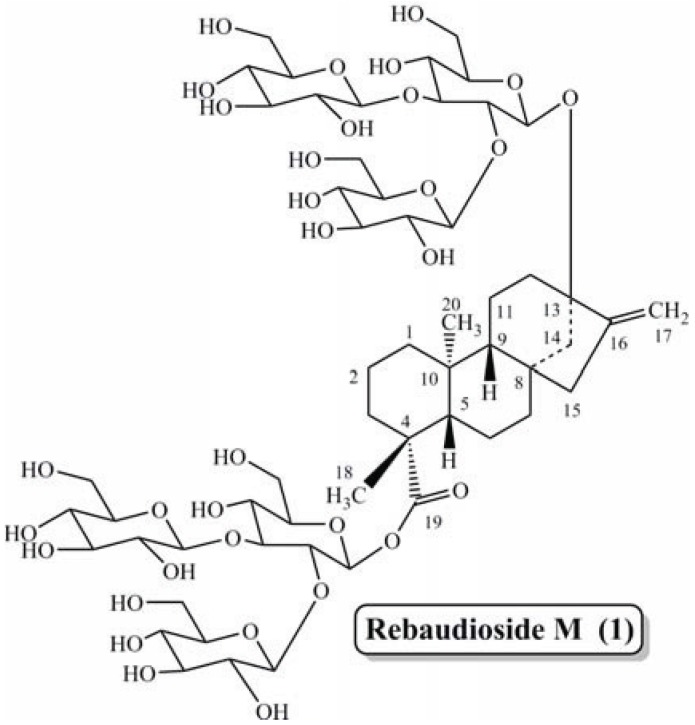
Structure of rebaudioside M (**1**).

*Stevia rebaudiana* Bertoni is a perennial shrub of the *Asteraceae* (*Compositae*) family native to certain regions of South America. Extracts of the leaves have been traditionally used for hundreds of years in Paraguay and Brazil to sweeten local teas and medicines [13,14]. The plant is commercially cultivated in Japan, Singapore, Taiwan, Malaysia, South Korea, China, Israel, India, Brazil, Australia and Paraguay. The leaves of *S. rebaudiana* contain several naturally sweet steviol glycoside such as steviolbioside, stevioside, rebaudioside A–F, dulcoside A and rubusoside [15,16,17,18,19,20]. Most recently we reported the isolation (from *S. rebaudiana* Bertoni), characterization and sensory evaluation for sweetness properties of rebaudioside M (also known as rebaudioside X) (**1**) (Figure 1) [21,22]. Recently Rebaudioside M has received a Letter of No Objection concerning its Generally Recognized as Safe (GRAS) status from the U.S. FDA [23].

**Scheme 1:**
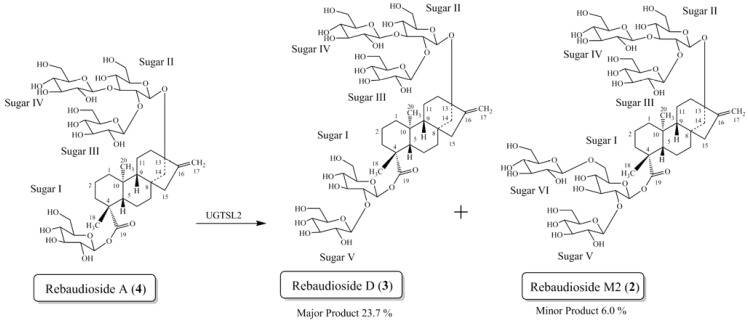
Bioconversion of rebaudioside A (**4**) to rebaudioside D (**3**) and minor product rebaudioside M2 (**2**).

**Figure 2:**
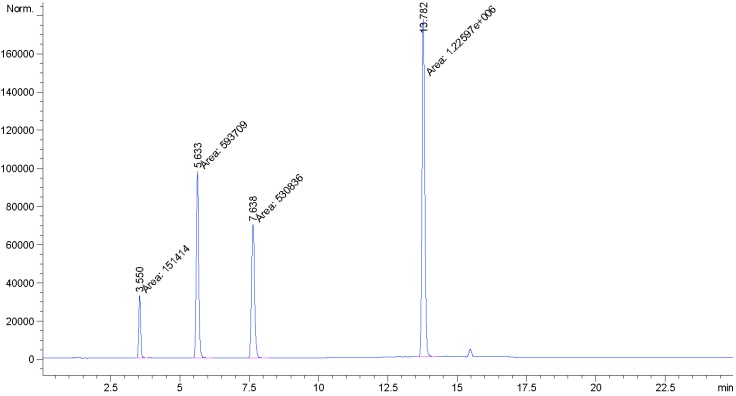
HPLC Chromatograph for the bioconversion of rebaudioside A (**4**) to rebaudioside D (**3**) and minor product rebaudioside M2 (**2**).

In the present study we report the isolation and characterization of a novel isomer of rebaudioside M, also called rebaudioside M2 (**2**) from the bioconversion reaction of rebaudioside A (**4**) to rebaudioside D (**3**) (Scheme 1). The bioconversion reaction mixture was analyzed by HPLC on Phenomenex Kinetex C18 100A (Phenomenex, Torrance, CA, USA), 4.6 mm × 150 mm, 2.6 µm; Column Temp: 55 °C; Mobile Phase A: 0.1% HCOOH in water; Mobile Phase B: Acetonitrile (MeCN); Flow Rate: 1.0 mL/min; Injection volume: 2 µL. Detection was by UV (210 nm) and MSD (SIM). Gradient: 0–8.5 min (75A:25B), 10.0 min (71A:29B), 10.0–16.5 (70A:30B), 25 min (70A:30B). Rebaudioside M2 was determined as a minor product (6.0% based on integrated area percent of the HPLC-MS chromatograph) (Figure 2).

**Table:** 

**Compound**	**Retention Time**	**MW**	**Peak Area**
Rebaudioside M2 (**2**)	3.550	1291	151,414
Rebaudioside D (**3**)	5.633	1129	593,709
Unknown I	7.638	1129	530,836
Rebaudioside A (**4**)	13.782	967	1,225,970

The structure of rebaudioside M2 (**2**) was determined by 1D and 2D NMR experiments together with mass spectral data. Further, a detailed NMR study was performed with rebaudioside M (Reb M) (**1**), rebaudioside D (Reb D) (**3**) and mixtures of rebaudioside M and rebaudioside D (all isolated from *S. rebaudiana* Bertoni) and the data compared with that previously reported for SG17 isolated from *S. rebaudiana* Morita [24,25].

## 2. Results and Discussion

### 2.1. Rebaudioside M2 (**2**)

To our knowledge this is the first report of isolation and complete characterization using NMR (^1^H, ^13^C, COSY, HSQC-DEPT, HMBC, 1D-TOCSY and NOESY) (Supplementary, Figure S1) and high resolution mass spectral data of rebaudioside M2 (**2**). Compound **2** was isolated as a white powder and accurate mass measurement using High Resolution Mass Spectrometry (HRMS) provided the exact mass *m/z* of 1289.5299, [M–H]^−^, in it’s negative ESI-TOF mass spectrum corresponding to a molecular formula of C_56_H_90_O_33_.

Examination of the 1D and 2D NMR data indicated the presence of a glycoside structure with a central diterpene core for compound **2**. The ^1^H-NMR spectrum and the HSQC-DEPT data of rebaudioside M2 (**2**) indicated the presence of two methyl singlets at δ 1.29 and 0.92, two olefinic proton singlets corresponding to an exocyclic double bond at δ 4.98 and at 5.16, nine methylene and two methine protons between δ 0.92–2.28 characteristic for the *ent*-kaurane diterpenoid isolated from other *Stevia* extracts [5,6,7,8,9,10,11,12]. The *ent-*kaurane diterpenoid aglycone central core was supported by ^1^H-^1^H COSY correlations of H-1/H-2; H-2/H-3; H-5/H-6; H-6/H-7; H-9/H-11; H-11/H-12 and ^1^H-^13^C HMBC correlations of H-5/C-1, C-20; H-9/C-1, C-7, C-14; H-17/C-13; H-18/C-3, C-5, C-19 and H-20/C-1, C-5, C-9, C-10. The complete ^1^H and ^13^C assignments of the central diterpene core are provided in Table 1 (positions 1–20).

**Figure 3:**
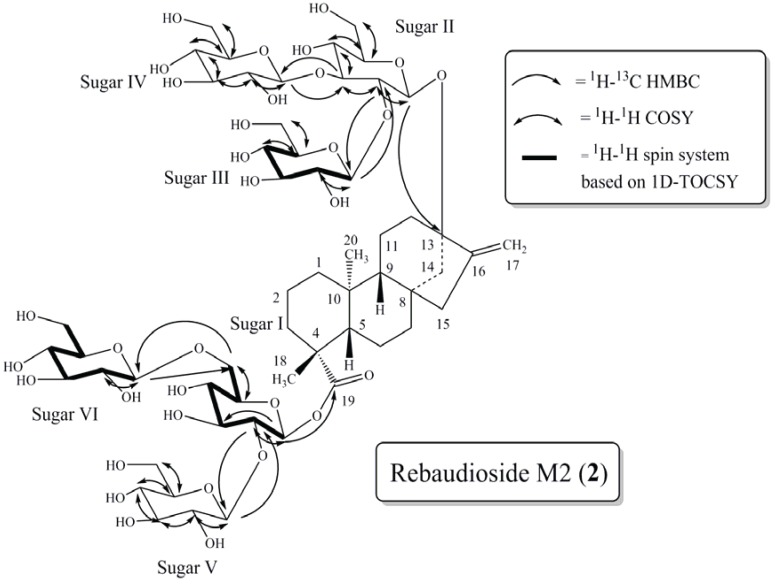
Key COSY, HMBC and 1D-TOCSY correlations of rebaudioside M2 (**2**).

Correlations observed in the NOESY spectrum were used to assign the relative stereochemistry of the central diterpene core. In the NOESY spectrum, NOE correlations were observed between H-14 and H-20 indicating that H-14 and H-20 are on the same face. Additionally, NOE correlations were observed between H-5 and H-9 but NOE correlations were not clearly observed between H-9 and H-14 indicating that H-5 and H-9 were on the opposite face of the rings compared to H-20 and H-14 as presented in Figure 3. Due to data overlap NOE correlations between H-5 and H-18 could not be confirmed, however the carbon chemical shifts support the relative stereochemistry as presented in Figure 3 [9,10,21]. These data thus indicated that the relative stereochemistry was retained during the glycosylation step.

**Table 1:** ^1^H-NMR (500 MHz, D_2_O) and ^13^C-NMR (125 MHz, D_2_O/TSP) assignments of rebaudioside M2 (**2**) ^a^.

Sugar	Position	^1^H-NMR	^13^C-NMR
	1	0.93 m,1.93 m	41.9
	2	1.49 m, 1.86 m	21.8
	3	1.16 m, 2.28 d (13.4)	39.8
	4	-	43.7
	5	1.24 d (12.1)	59.2
	6	1.73 m, 1.94 m	24.4
	7	1.49 m, 1.56 m	44.2
	8	-	46.9
	9	1.09 d (7.7)	55.5
	10	-	42.4
	11	1.66 m, 1.70 m	22.6
	12	1.60 m, 2.00 m	39.9
	13	-	90.9
	14	1.53 d (12.6), 2.21 d (13.6)	46.9
	15	2.15 d (17.2), 2.18 d (18.1)	49.4
	16	-	164.0
	17	4.98 s, 5.16 s	107.0
	18	1.29 s	31.0
	19	-	181.5
	20	0.92 s	19.1
I	1'	5.65 d (7.6)	95.5
	2'	3.96 m	80.5
	3'	3.89 m	79.0
	4'	3.71 m	71.5
	5'	3.73 m	79.0
	6'	4.00 m, 4.15 d (11.7)	70.9
II	1''	4.85 d (7.8)	98.4
	2''	3.75 m	81.7
	3''	3.98 m	88.0
	4''	3.54 m	71.3
	5''	3.96 m	80.5
	6''	3.45 m, 3.77 m	63.6
III	1'''	4.92 d (7.9)	104.9
	2'''	3.32 m	76.3
	3'''	3.51 m	78.8
	4'''	3.26 t (9.5)	73.3
	5'''	3.44 m	78.8
	6'''	3.75 m, 3.94 m	64.4
IV	1''''	4.84 * d (7.8)	105.0 *
	2''''	3.41 m	76.1
	3''''	3.46 m	78.8
	4''''	3.45 m	72.5
	5''''	3.75 m	81.7
	6''''	3.55 m, 3.78 m	65.8
V	1'''''	4.83 * d (8.0)	105.3 *
	2'''''	3.32 m	78.5
	3'''''	3.51 m	78.7
	4'''''	3.38 m	72.9
	5'''''	3.55 m	78.8
	6'''''	3.76 m, 3.97 m	63.6
VI	1''''''	4.50 d (7.9)	105.7
	2''''''	3.33 m	78.1
	3''''''	3.49 m	78.6
	4''''''	3.45 m	72.3
	5''''''	3.48 m	78.8
	6''''''	3.92 m, 3.94 m	64.1

^a^ Assignments were made on the basis of COSY, HSQC-DEPT, HMBC, and 1D-TOCSY correlations; chemical shift (δ) values are in ppm; and coupling constants are in Hz; * ^1^H and ^13^C values can be exchangeable.

The ESI HRMS/MS spectrum of compound **2** showed ions at *m/z* 803.3688, 641.3165, 479.2633 and 317.2082 corresponding to the loss of three hexose sugars followed by sequential loss of three additional hexose sugar moieties from it’s [M–H]^−^ ion at *m/z* 1289.5. The presence of six anomeric protons evident from the ^1^H and ^1^H-^13^C HSQC-DEPT spectra [δ_H_ 5.65 (δ_C_ 95.5), 4.92 (δ_C_ 104.9), 4.85 (δ_C_ 98.4), 4.83/4.84 (δ_C_ 105.0/105.3), 4.83/4.84 (δ_C_ 105.3/105.0) and 4.50 (δ_C_ 105.7)], confirmed the presence of six sugar units in the structure. The complete assembly of the glycoside structure was done on the basis of correlations observed in the 2D and 1D-TOCSY NMR data. Thus, long range ^1^H-^13^C correlations observed in the HMBC experiment from the anomeric proton at δ_H_ 5.65 to a carbonyl carbon at δ_C_ 181.5 (C-19) allowed its assignment as the anomeric proton (H-1') of sugar I. Similarly, HMBC correlation from the anomeric proton observed at δ_H_ 4.85 to a quaternary carbon at δ_C_ 90.9 (C-13) allowed it to be assigned as the anomeric proton (H-2'') of sugar II (Figure 3).

Further analysis of the 1D and 2D NMR data allowed the assignment of the remaining four sugars in **2**. The relatively downfield chemical shift of C-2' (δ_C_ 80.5) and C-6' (δ_C_ 70.9) in sugar I suggested a 2,6-branched-d-glucotriosyl substituent at C-19. Long range ^1^H-^13^C correlations observed in the HMBC experiment from the anomeric proton observed at δ_H_ 4.83/4.84 (H-1''''') to the carbon at δ_C_ 80.5 (C-2') and from H-2' at δ_H_ 3.96 to an anomeric carbon at δ_C_ 105.3/105.0 (C-1''''') confirmed the substitution at C-2' in sugar I. Additionally, HMBC correlations observed from the anomeric proton at δ_H_ 4.50 (H-1'''''') to the carbon at δ_C_ 70.9 (C-6') and from the methylene protons of sugar I at δ_H_ 4.00 and 4.15 to the anomeric carbon (δ_C_ 105.7) of C-1'''''' confirmed the presence of a 1**→**6 sugar linkage between sugar VI and sugar I.

The remaining two glucose moieties were assigned in a similar manner. The relatively downfield chemical shift of C-2'' (δ_C_ 81.7) and C-3'' (δ_C_ 88.0) in sugar II suggested a 2,3-branched-d-glucotriosyl substituent at C-13. Long range ^1^H-^13^C correlations observed in the HMBC experiment from the anomeric proton observed at δ_H_ 4.92 (H-1''') to the carbon at δ_C_ 81.7 (C-2'') and from H-2'' at δ_H_ 3.75 to an anomeric carbon at δ_C_ 104.9 (C-1''') confirmed the sugar substitution at C-2' in sugar II. Similarly, the sugar substituent at C-3'' in sugar II was also corroborated by HMBC correlations observed from the anomeric proton at δ_H_ 4.84/4.83 (H-1'''') to the carbon at δ_C_ 88.0 (C-3'') and from H-3'' at δ_H_ 3.98 to the anomeric carbon (δ_C_ 105.3/105.0) of sugar IV confirmed the presence of a 1**→**3 sugar linkage between sugar IV and sugar II.

The large coupling constants observed for the anomeric protons of the glucose moieties at δ_H_ 5.65 (d, *J* = 7.6 Hz), 4.92 (d, *J* = 7.9 Hz) and 4.50 (d, *J* = 7.9 Hz) suggested their β-orientation. While the remaining three anomeric protons, at δ_H_ 4.83 (d, *J* = 7.8 Hz), 4.84 (d, *J* = 7.8 Hz) and 4.85 (d, *J* = 8.0 Hz), were not completely resolved their apparent coupling constants also indicated β-orientation.

The ^1^H and ^13^C chemical shifts for the glycoside at C-13 and C-19 are found in Table 1 and a summary of the key HMBC, COSY, and 1D-TOCSY correlations used to assign the glycoside are provided in Figure 3.

Thus the structure of rebaudioside M2 (**2**), containing a relatively rare 1**→**6 sugar linkage, was established as (13-[(2-*O*-β-d-glucopyranosyl-3-*O*-β-d-glucopyranosyl-β-d-glucopyranosyl)oxy] *ent*-kaur-16-en-19-oic acid-[(2-*O*-β-d-glucopyranosyl-6-*O*-β-d-glucopyranosyl-β-d-glucopyranosyl) ester].

### 2.2. NMR Study of Rebaudioside M, Rebaudioside D and SG17

During isolation (HPLC-MS) of compounds **1** and **2** it was revealed that more than one peak provided the molecular weight of 1290, and thus NMR studies were critical for complete structure determination. To determine the complete structure of rebaudioside M2 (**2**) we compared the NMR spectral data of rebaudioside M (**1**) (reported in Prakash *et al.* 2013) [21] and the NMR spectral data for SG17 (reported in Ohta *et al.* 2010) [24]. Unfortunately rebaudioside M2 (**2**) was not soluble in any of the solvents screened except for D_2_O and thus its data could not be directly compared to previously reported data which were acquired in different solvent systems. This led to further examination of the two reported NMR spectral data for rebaudioside M, Ohta *et al.* [21] and Prakash *et al.* [24], and noticeable differences in spectral data, acquired in the same solvent system (Pyridine-*d*_5_ + TMS) were observed.

Thus, a series of NMR experiments including ^1^H, ^13^C, ^1^H-^1^H COSY, ^1^H-^13^C HSQC-DEPT, and ^1^H-^13^C HMBC were performed in pyridine-*d*_5_ + TMS (Supplementary, Figure S2) to allow assignment of >95% rebaudioside M (**1**) in this solvent system. The ^1^H- and ^13^C-NMR data of >95% rebaudioside M (**1**) in pyridine-*d*_5_ + TMS, were compared to the data of SG17 (isolated from *S. rebaudiana* Morita) in pyridine-*d*_5_ + TMS, which was previously reported by Ohta *et al.* [24]. As presented in Table 2 and Table 3, more than half of the reported ^1^H and ^13^C chemical shifts of SG17 are not consistent with the ^1^H and ^13^C chemical shifts of compound **1** indicating that the data presented for SG17 by Ohta *et al.* [24] are most likely for a mixture of steviol glycosides. Therefore, NMR analysis of related samples such as >95% rebaudioside D (**3**) (Exp. 2), 80% rebaudioside M (**1**) (Exp. 5), and mixture of >95% rebaudioside M and >95% rebaudioside D (Exp. 3 and Exp. 4) were carried out (Supplementary, Figures S3–S6) to compare their data to the data of SG17. The ^13^C- and ^1^H-NMR assignments for compounds **1** and **3** in experiments 3 and 4 (Table 2 and Table 3) were confirmed on the basis of HSQC-DEPT and HMBC data. All compounds used in this NMR study were isolated from *S. rebaudiana* Bertoni.

The data presented by an asterisk for SG17 in Table 2 (with the exception of assignments for C9, C11 and C12) differ by about 1 ppm from the data of rebaudioside M (**1**) clearly indicate that these data do not belong to rebaudioside M. Instead, the NMR data of SG17 presented by an asterisk are consistent with the data of rebaudioside D (**3**). Some of the data of SG17, however, match with the data of compound **1** but some do not match with either the data of rebaudioside M (**1**) or rebaudioside D (**3**). Similarly, in the ^1^H-NMR spectrum of SG17 (Table 3), the data of sugar III H-1' and sugar IV H-1' are most likely swapped, otherwise are consistent with the data of rebaudioside D (**3**).

Furthermore, as reported in Ohta *et al.* [24], the structure characterization of SG17 was performed in part by partial hydrolysis of the glycoside rather than using the modern 1D and 2D NMR techniques which are very commonly and widely used methods for structure elucidation of unknown compounds such as rebaudioside M. As reported in Morita *et al.* [25], rebaudioside M and rebaudioside D were separated by HPLC as a combined peak, and the rebaudioside M structure was deduced from mass spectrometry data, rather than using purified rebaudioside M for characterization by the modern 1D and 2D NMR techniques. Further, complete ^1^H- and ^13^C-NMR spectral assignments of the glycoside SG17 is not provided in the paper or patent application [24,25]. For a compound as complex as rebaudioside M (**1**), 2D NMR data is needed to establish the specific sugar linkages and complete assignment of the structure, however in the publications of Ohta *et al.* [24], and Morita *et al.* [25] there was no indication of any 2D NMR or advance 1D NMR experiments utilized. In the absence of such NMR data, complex structures such as rebaudioside M can be misidentified.

In summary, based on the numerous NMR studies it was inferred that SG17 could be a mixture of rebaudioside D (**3**), rebaudioside M (**1**), and possibly related steviol glycosides, with rebaudioside D (**3**) as the major compound. This work not only uncovers a novel steviol glycoside (**2**), containing a relatively rare 1**→**6 sugar linkage, it also illustrates how critical 1D and 2D NMR techniques are when identifying complex steviol glycosides.

**Table 2:** Comparison of ^13^C-NMR data (125 MHz, pyridine-*d*_5_ + TMS) of Rebaudioside M (**1**), Rebaudioside D (**3**), mixtures of (**1**) and (**3**) and SG17 (75 MHz, pyridine-*d*_5_ + TMS).

Position No.	Exp. 1 ^13^C Assignment for >95% Reb M (1) in Pyr-*d_5_* + TMS	Exp. 2 ^13^C Assignment for >95% Reb D (3) in Pyr-*d*_5_ + TMS	Exp. 3 ^13^C Assignment for (1) 82% + (3) (18%) in Pyr-*d_5_* + TMS		Exp. 4 ^13^C Assignment for (3) 82% + (1) 18% in Pyr-*d_5_* + TMS		Exp. 5 ^13^C Assignment for 80% Reb M (1) in Pyr-*d_5_* + TMS	^13^C Assignment [24] in Pyr-*d_5_* + TMS for SG17
			Reb M	Reb D	Reb D	Reb M		
1	40.3	40.6	40.3	40.6	40.6	40.3	40.3	40.5
2	19.6	20.0	19.6	20.0	20.0	19.6	19.6	19.8
3	38.4	37.8	38.4	37.8	37.8	38.4	38.4	38.3
4	44.2	44.3	44.2	44.3	44.3	44.3	44.2	44.3
5	57.3	57.4	57.3	57.4	57.4	57.3	57.3	57.3
6	23.4	22.2 *	23.4	22.2	22.2	23.4	23.4	22.2 *
7	42.5	42.2	42.5	42.2	42.2	42.5	42.5	41.1 *
8	41.2	41.8	41.1	41.8	41.8	41.1	41.2	42.1 *
9	54.2	53.9 *	54.2	53.9	53.9	54.2	54.2	53.9 *
10	39.7	39.7	39.7	39.7	39.7	39.7	39.7	39.7
11	20.2	20.5 *	20.2	20.5	20.5	20.2	20.2	20.5 *
12	38.4	37.8 *	38.4	37.8	37.8	38.4	38.4	37.8 *
13	87.6	86.7 *	87.6	86.7	86.7	87.5	87.6	86.6 *
14	43.3	44.1 *	43.3	44.1	44.1	43.3	43.3	44.2 *
15	46.5	47.7 *	46.5	47.7	47.7	46.4	46.5	47.6 *
16	153.2	154.0 *	153.3	154.0	154.0	153.3	153.2	154.0 *
17	104.9	104.8	104.9	104.8	104.8	104.9	104.9	104.0
18	28.2	29.2 *	28.2	29.2	29.2	28.2	28.2	29.3 *
19	176.9	175.8 *	176.9	175.8	175.8	176.8	176.9	175.7 *
20	16.7	16.8	16.7	16.8	16.8	16.7	16.7	16.8
C-1'	94.9	93.6 *	94.9	93.6	93.6	94.9	94.9	93.6 *
C-1''	96.2	97.8 *	96.2	97.8	97.8	96.2	96.2	97.8 *
C-1'''	104.7	104.5	104.8	104.5	104.5	104.8	104.7	104.6
C-1''''	103.9	104.7 *	103.9	104.5	104.7	103.9	103.9	104.7 *
C-1'''''	104.1	105.7	104.1	105.7	105.7	104.2	104.1	104.1
C-1''''''	104.1	NA	104.1	NA	NA	104.1	104.1	105.1

Reb D = Rebaudioside D; Reb M = Rebaudioside M; NR: Not reported; NA: Not applicable for Reb D; * = Similar spectral data.

**Table 3:** Comparison of ^1^H-NMR data (500 MHz, pyridine-*d*_5_ + TMS) for **1**, **3** and SG17 (300 MHz, pyridine-*d*_5_ + TMS).

Position No.	Exp. 1 ^1^H Assignment for >95% Rebaudioside M (1) in Pyr-*d_5_* + TMS	Exp. 2 ^1^H Assignment for >95% Rebaudioside D (3) in Pyr-*d_5_* + TMS	^1^H Assignment [24] in Pyr-*d_5_* + TMS for SG17
1	0.77 t (11.5)	0.75 t (11.4)	NR
	1.78 m	1.72 m	
2	1.39 m	1.44 m	NR
	2.27 m	2.16 m	
3	1.04 m	1.10 m	NR
	2.32 d (13.7)	2.72 d (13.0)	
4	-	-	NR
5	1.08 d (13.4)	1.00 d (13.0)	NR
6	2.25 m	1.90 m	NR
	2.42 q (12.9)	2.18 m	
7	1.44 m	1.29 m	NR
	1.82 m	1.42 m	
8	-	-	NR
9	0.93 d (7.8)	0.90 d (6.9)	NR
10	-	-	NR
11	1.67 m	1.67 m	NR
	1.78 m	1.70 m	
12	1.88 m	1.91 m	NR
	2.74 m	2.24 m	
13	-	-	NR
14	2.04 m	1.79 d (12.3)	NR
	2.75 m	2.53 d (10.9)	
15	1.91 d (17.7)	2.02 m	NR
	2.06 m	2.06 m	
16	-	-	NR
17	4.92 s	5.05 s	NR
	5.71 s	5.68 s	
18	1.35 s	1.42 s	NR
19	-	-	NR
20	1.39 s	1.17 s	NR
H-1'	6.40 d (8.3)	6.32 d (7.6) *	6.33 d (7.4) *
H-1''	5.47 d (7.9)	5.10 d (7.5) *	5.07 d (7.8) *
H-1'''	5.50 d (7.5)	5.58 d (7.8)	5.41 d (7.8)
H-1''''	5.47 d (7.9)	5.40 d (7.9)	5.59 d (7.5)
H-1'''''	5.82 d (7.4)	5.48 d (7.7) *	5.48 d (7.7) *
H-1''''''	5.33 d (8.0)	NA	5.33 d (7.7)

NR: Not reported; NA: Not applicable for Reb D; * = Similar spectral data.

## 3. Experimental

### 3.1. General Experimental Procedures for Rebaudioside M2 (**2**)

#### 3.1.1. Isolation and Purification

Preliminary HPLC analyses of samples were performed using a Waters 2695 Alliance System (Waters Corp., Milford, MA, USA) equipped with a Waters 2996 Photodiode Array (PDA, Waters Corp.) and Dionex Corona Charged Aerosol (CAD Plus, Dionex, Sunnyvale, CA, USA) detectors by the following method: Phenomenex Synergi Hydro-RP, 4.6 × 250 mm, 4 µm (p/n 00G-4375-E0); Column Temp: 55 °C; Mobile Phase A: 0.0284% NH_4_OAc and 0.0116% HOAc in water; Mobile Phase B: Acetonitrile (MeCN); Flow Rate: 1.0 mL/min; Injection volume: 10 µL. Detection was by UV (210 nm) and CAD. Gradient: 0–8.5 min (75A:25B), 10 min (71A:29B), 16.5 min (70A:30B), 18.5–24.5 (66A:34B), 26.5–29.0 min (48A:52B), 31–37 min (30A:70B), 38 min (75A:25B).

Analyses of the semi-preparative purification fractions were performed using the following method: Waters Atlantis dC18, 4.6 × 100 mm, 5 μm (p/n 186001340); Mobile Phase A: 25% MeCN in water; Mobile Phase B: 30% MeCN in water; Flow rate: 1.0 mL/min; Injection volume: 10 μL, Detection by CAD. Gradient: 0–5 min (100A), 20 min (20A:80B), 25 min (20A:80B), 30 min (100A).

LC-MS: Preliminary analysis of the steviol glycoside mixture from *S. rebaudiana* Bertoni was carried out on either a Waters Auto Purification HPLC/MS System with a Waters 3100 Mass Detector or a Waters 2695 Alliance System equipped with a Waters 2996 PDA and Waters QToF Micro detectors operating in negative ion mode. Analysis of the sample was performed using the following method: Phenomenex Synergi Hydro-RP, 4.6 × 250 mm, 4 µm (p/n 00G-4375-E0); Column Temp: 55 °C; Mobile Phase A: 0.0284% NH_4_OAc and 0.0116% HOAc in water; Mobile Phase B: MeCN; Flow Rate: 1.0 mL/min; Injection volume: 10 µL. Detection was by UV (210 nm), and MSD (−ESI *m*/*z* 500–2000). Gradient: 0–8.5 min (75A: 25B), 10 min (71 A:29B), 16.5 min (70A: 30B), 18.5–24.5 min (66A:34B), 26.5–29.0 min (48A:52B), 31–37 min (30A:70B), 38 min (75A:25B).

Isolation of **2** by HPLC: The purification was performed in two chromatographic steps. The first method used for the semi-preparative purification is summarized below. Column: Waters Atlantis dC18, 30 × 100 mm, 5 µm (p/n 186001375); Mobile Phase A: 25% MeCN in water; Mobile Phase B: 30% MeCN in water; Flow Rate: 45 mL/min; Injection load: 160 mg dissolved in 20 mL of water. Detection was by UV (205 nm). Gradient: 0–5 min (100A), 20 min (20A:80B), 25 min (20A:80B), 30 min (100A). The secondary purification used the same column and conditions, but isocratic mobile phase: 20% MeCN in water.

#### 3.1.2. Mass Spectrometry

The ESI-TOF mass spectra and MS/MS data were generated by a Waters QTof Premier mass spectrometer (Waters Corp., Manchester, UK) equipped with an electrospray ionization source. Samples were analyzed by negative ESI. Samples were diluted with H_2_O:MeCN (1:1) by 50-fold and introduced via infusion using the onboard syringe pump.

#### 3.1.3. Nuclear Magnetic Resonance

The sample of Rebaudioside M2 (**2**) (~1.0 mg in 150 µL of D_2_O) was prepared and NMR data were acquired on Bruker Avance 500 MHz instrument (Bruker BioSpin Corp., Billerica, MA, USA) with a 2.5 mm inverse detection probe and 5 mm broad band probe. The ^1^H-NMR and ^13^C-NMR spectra were referenced to the residual solvent signal HDO (δ_H_ 4.79 ppm) and TSP (δ_C_ 0.00 ppm), respectively. 

*NMR Studies for rebaudioside M* (**1**), *rebaudioside D* (**3**), *and* (**1**) *+* (**3**) *mixtures*

The sample of >95% Rebaudioside M (**1**) (10.6 mg in 0.2 mL of pyridine-*d*_5_ + TMS), 80% Rebaudioside M (**1**) (10.1 mg in 0.2 mL of pyridine-*d*_5_ + TMS), >95% Rebaudioside D (**3**) (10.7 mg in 0.2 mL of pyridine-*d*_5_ + TMS), 82% of >95% Rebaudioside M (**1**) and 18% of >95% Rebaudioside D (**3**) (8.2 mg and 1.8 mg, respectively in 0.2 mL of pyridine-*d*_5_ + TMS), and 18% of >95% Rebaudioside M (**1**) and 82% of >95% Rebaudioside D (**3**) (1.8 mg and 8.2 mg, respectively in 0.2 mL of pyridine-*d*_5_ + TMS), were prepared and NMR data were acquired on Bruker Avance 500 MHz instrument utilizing 5 mm probe except for >95% Rebaudioside M (**1**) and >95% Rebaudioside D (**3**), for which 2.5 mm and 5 mm probes were used. The spectra were referenced to the residual solvent signals, δ_H_ 0.00, δ_C_ 0.0 for TMS present in pyridine-*d*_5_ + TMS, chemical shifts (δ_H_ and δ_C_ are given in ppm, and coupling constant reported in Hertz.

### 3.2. Material Sources

For the NMR studies rebaudioside M (**1**), rebaudioside D (**3**) and mixtures of (**1**) and (**3**) were all isolated from *S. rebaudiana* Bertoni extract (PureCircle Lot# C4-001-1012-0001, PureCircle Ltd., Bandar Enstek, Negeri Sembilan, Malaysia).

### 3.3. Bioconversion Reaction

Rebaudioside M2 (**2**) was isolated from bioconversion reaction of rebaudioside A (**4**) to rebaudioside D (**3**) by a proprietary glucosyltransferase from PureCircle Ltd. *In vivo* production of glycosylation enzymes were expressed in yeast.

Rebaudioside A to rebaudioside D conversion with glucosyltransferase UGTSL2 experiment condition are as follows: 430 μL of a reaction mixture containing 0.5 mM rebaudioside A, 3 mM MgCl_2_, 50 mM sodium phosphate buffer at pH 7.2 and 2.5 mM of UDP-Glucose was added to a 1.5 mL sterile microtube. 52 μL of the enzyme expressed medium was added and the resulting mixture was allowed to react at 30 °C for 24 h. 125 μL samples were taken after 2 h, 16 h, and 24 h and added to a 115 μL of 60% methanol and 10 μL of 2 N sulfuric acid. The quenched sample was centrifuged at 18,000× *g* for 2 min at room temperature. 200 μL was transferred to a HPLC vial and analyzed by the method described below.

HPLC analyses of samples were performed using an Agilent 1200 series HPLC system equipped with a binary pump (G1312B), autosampler (G1367D), thermostatted compartment (G13136B) and DAD detector (G1315C), connected with Agilent 6110 A MSD, and interfaced with “LC/MSD Chemstation” software. The conditions used were Phenomenex Kinetex, 2.6 μm C18 100A, 4.6 mm × 150 mm, 2.6 μm; Column Temp: 55 °C; Mobile Phase A: 0.1% formic acid in water; Mobile Phase B: Acetonitrile (MeCN); Flow Rate: 1.0 mL/min; Injection volume: 2 µL. Detection was by DAD (210 nm) and MSD (Scan and SIM mode, ES-API, negative polarity). Gradient: 0–8.5 min (75A:25B), 10 min (71A:29B), 16.5 min (70A:30B).

### 3.4. General Experimental Procedures for Rebaudioside D (**3**)

Rebaudioside D (**3**), bioconversion desired product, was isolated and characterized by NMR and MS which allowed a full assignment confirmation.

#### 3.4.1. Isolation and Purification

Preliminary HPLC analyses of samples were performed using a Waters 2695 Alliance System using the following method: Phenomenex Synergi Hydro-RP, 4.6 × 250 mm, 4 µm (p/n 00G-4375-E0); Column Temp: 55 °C; Mobile Phase A: 0.0284% NH_4_OAc and 0.0116% HOAc in water; Mobile Phase B: Acetonitrile (MeCN); Flow Rate: 1.0 mL/min; Injection volume: 10 µL. Detection was by UV (210 nm) and CAD. Gradient: 0–8.5 min (75A:25B), 10 min (71A:29B), 16.5 min (70A:30B), 18.5–24.5 (66A:34B), 26.5–29.0 min (48A:52B), 31–37 min (30A:70B), 38 min (75A:25B).

Analyses of the bioconversion steviol glycoside mixture were performed by the following method: Waters AutoPurification HPLC/MS System with a Waters 3100 mass Decector operating in the negative ion mode. Analysis of the sample was performed using the following method: Phenomenex Synergi Hydro-RP, 4.6 × 250 mm, 4 µm (p/n 00G-4375-E0); Column Temp: 55 °C; Mobile Phase A: 0.0284% NH_4_OAc and 0.0116% HOAc in water; Mobile Phase B: MeCN; Flow Rate: 1.0 mL/min; Injection volume: 10 µL. Detection was by UV (210 nm), and MSD (−ESI *m*/*z* 500–2000). Gradient: 0–8.5 min (75A:25B), 10 min (71A:29B), 16.5 min (70A:30B), 18.5–24.5 (66A:34B), 26.5–29.0 min (48A:52B), 31–37 min (30A:70B), 38 min (75A:25B).

Isolation of **3** by HPLC: The secondary purification used the same column and conditions, but isocratic mobile phase: 20% MeCN in water. The purification conditions are summarized below. Column: Waters Atlantis T3, 10 × 250 mm, 5 µm (p/n 186003694); Mobile Phase A: DI Water; Mobile Phase B: Acetonitrile; Flow Rate: 5 mL/min; Injection load: 1mL of 74 mg/mL solution. Detection was by mass spectrometry, single ion response detection. Gradient: 0 min (70A:30B), 15 min (60A:40B), 25 min (46A:54B), 30 min (20A:80B), 30.1–40 min (70A:30B).

#### 3.4.2. Mass Spectrometry

The ESI-TOF mass spectra and MS/MS data were generated with a Waters Q-Tof Premier mass spectrometer equipped with an electrospray ionization source. Samples were analyzed by negative ESI. Samples were diluted with H_2_O:MeCN (1:1) by 50-fold and introduced via infusion using the onboard syringe pump.

#### 3.4.3. Nuclear Magnetic Resonance

The sample of rebaudioside D (**3**) was prepared by dissolving 10.7 mg in 200 µL of pyridine-*d*_5_ + TMS and NMR data were acquired on Bruker Avance 500 MHz instruments with a 2.5 mm inverse detection and 5 mm broad band probes. The ^1^H-NMR and ^13^C-NMR spectra were referenced to TMS resonance (δ_H_ 0.00 ppm and δ_C_ 0.00 ppm).

## 4. Conclusions

To the best of our knowledge this is the first report of full isolation and spectral characterization of (13-[(2-*O*-β-d-glucopyranosyl-3-*O*-β-d-glucopyranosyl-β-d-glucopyranosyl)oxy]*ent*-kaur-16-en-19-oic acid-[(2-*O*-β-d-glucopyranosyl-6-*O*-β-d-glucopyranosyl-β-d-glucopyranosyl)ester]), rebaudioside M2 (**2**), from a bioconversion reaction of rebaudioside A **(4**) to rebaudioside D (**3**). Rebaudioside M2 (**2**) possesses a 1**→**6 sugar linkage between sugar VI and sugar I, making its structural properties unique. Continued discovery in the area of novel steviol glycosides provides great opportunity to find novel sweeteners or sweetener enhancers that can improve sweet taste. In addition to the discovery of rebaudioside M2 (**2**), we have reiterated the importance of multiple 1D and 2D NMR techniques when identifying complex steviol glycosides. Thus extensive NMR studies of rebaudioside M (**1**), rebaudioside D (**3**), and mixtures of **1** and **3** led to the discovery that SG17 is not a single compound but is a mixture of rebaudioside D (**3**), rebaudioside M (**1**), and possibly other related steviol glycosides.

## Supplementary Files

Supplementary File 1 Supplementary Materials (PDF, 563 KB)
