# Supplementary material for: Isolation and Characterization of a Novel Rebaudioside M Isomer from a Bioconversion Reaction of Rebaudioside A and NMR Comparison Studies of Rebaudioside M Isolated from Stevia rebaudiana Bertoni and Stevia rebaudiana Morita
Source: Biomolecules. 2014 Mar 31;4(2):374–89. doi: 10.3390/biom4020374 (PMC4101487; doi:10.3390/biom4020374)
Supplement: Supplementary File 1 — Supplementary Materials (PDF, 563 KB) [file biomolecules-04-00374-s001.pdf]

Isolation and Characterization of a Novel Rebaudioside M Isomer from a Bioconversion Reaction of Rebaudioside A and NMR Comparison Studies of Rebaudioside M Isolated from *Stevia rebaudiana* Bertoni and *Stevia rebaudiana* Morita

Supplementary Materials

Figure S1. 1D and 2D NMR spectra of Rebaudioside M2 (**2**). (**A**) ^{1}H-NMR spectrum of **2**; (**B**) ^{13}C-NMR spectrum of **2**; (**C)** ^{1}H-^{1}H COSY spectrum of **2**; (**D**) ^{1}H-^{13}C HSQC-DEPT spectrum of **2**; (**E**) ^{1}H-^{13}C HMBC spectrum of **2**; (**F**) ^{1}H-^{1}H NOESY spectrum of **2**.

A

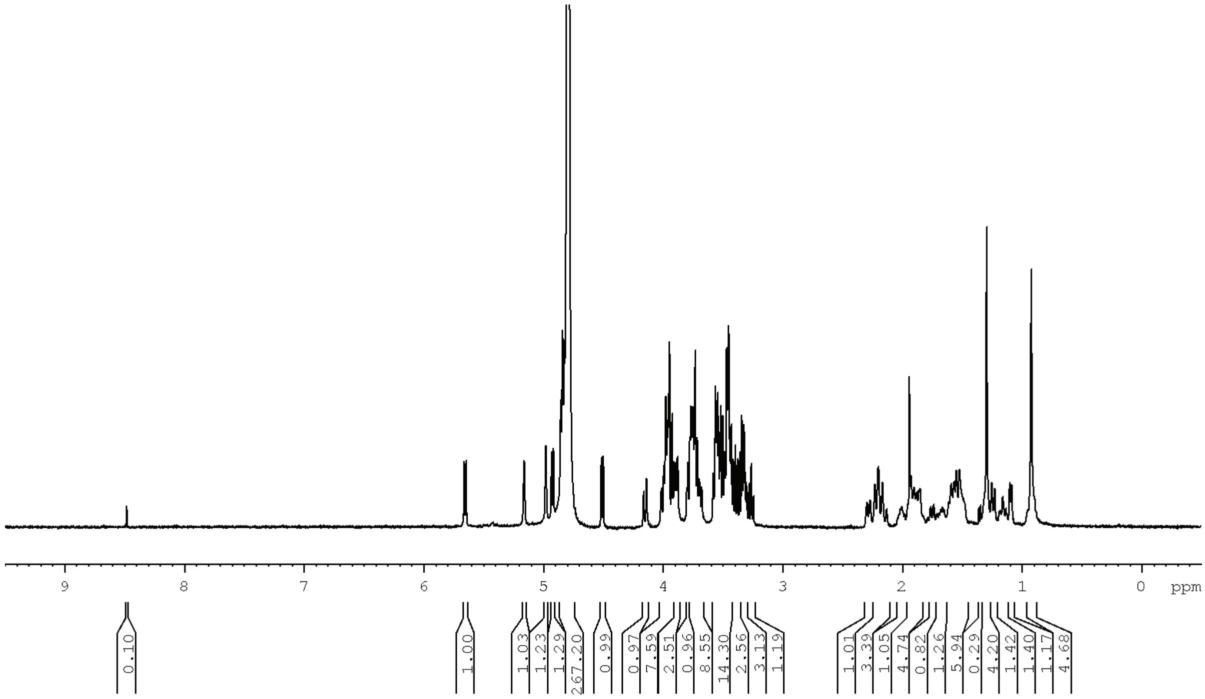

B

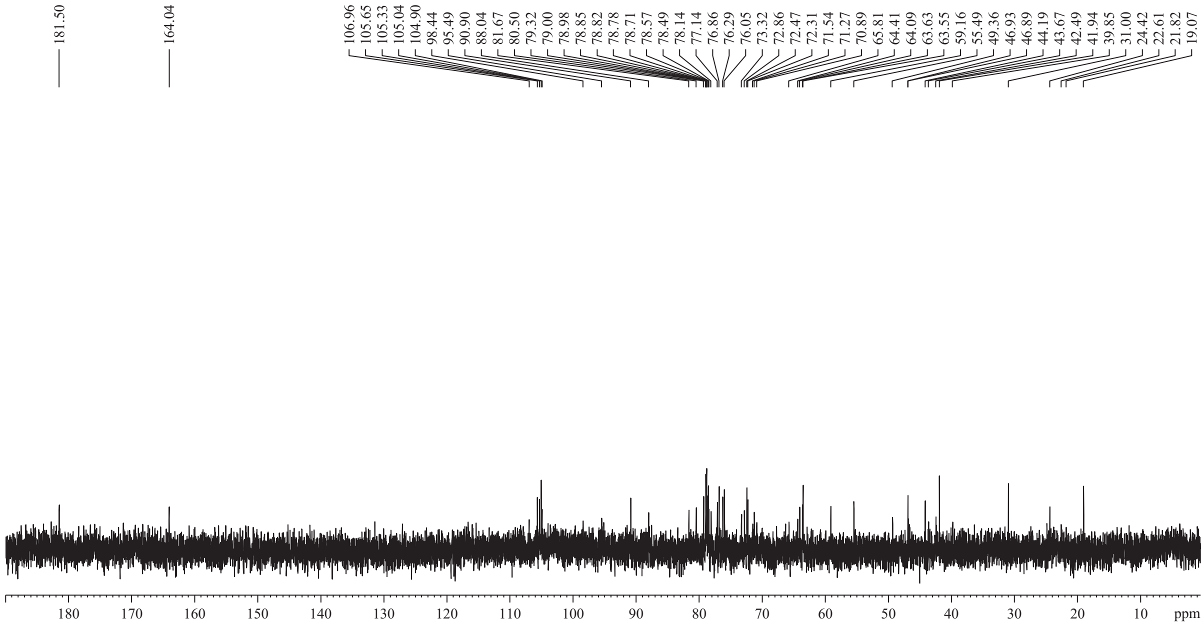

Figure S1. *Cont.*

C

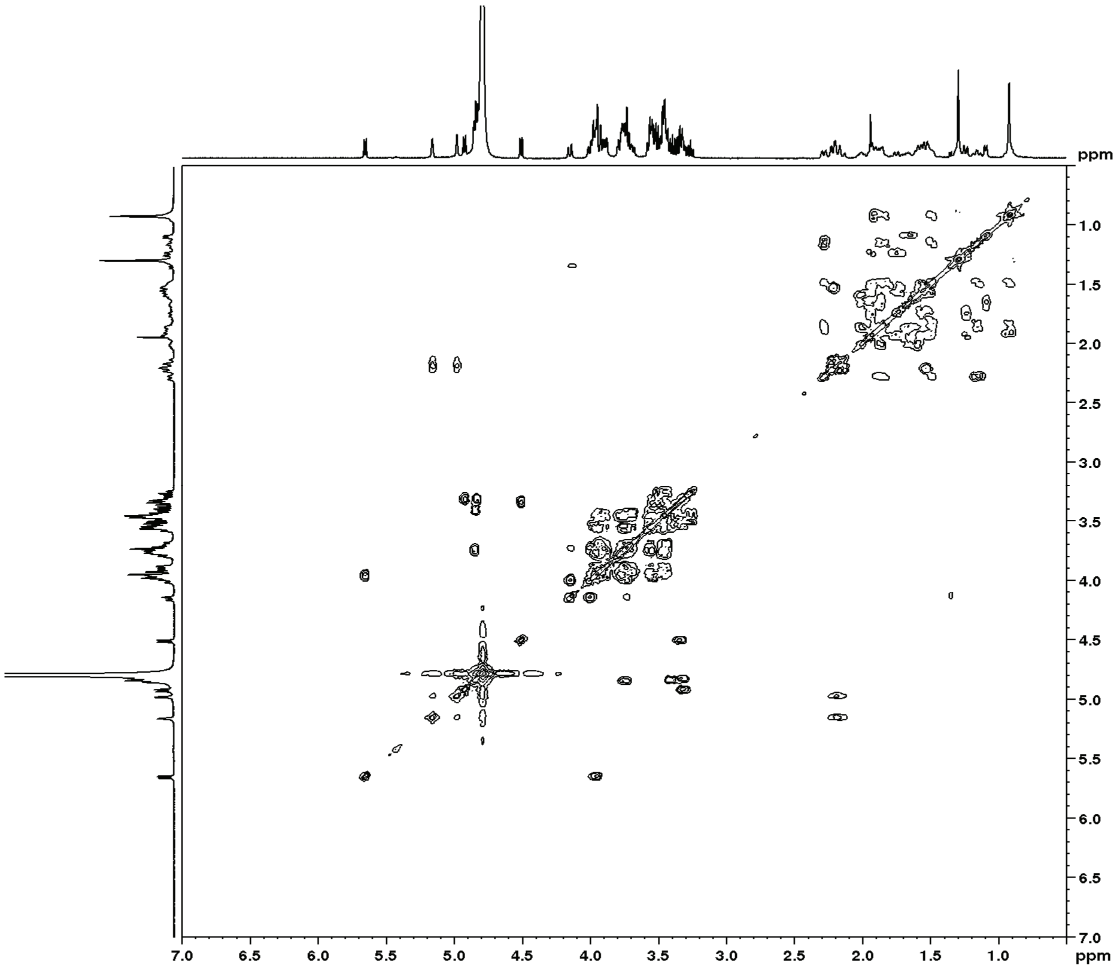

D

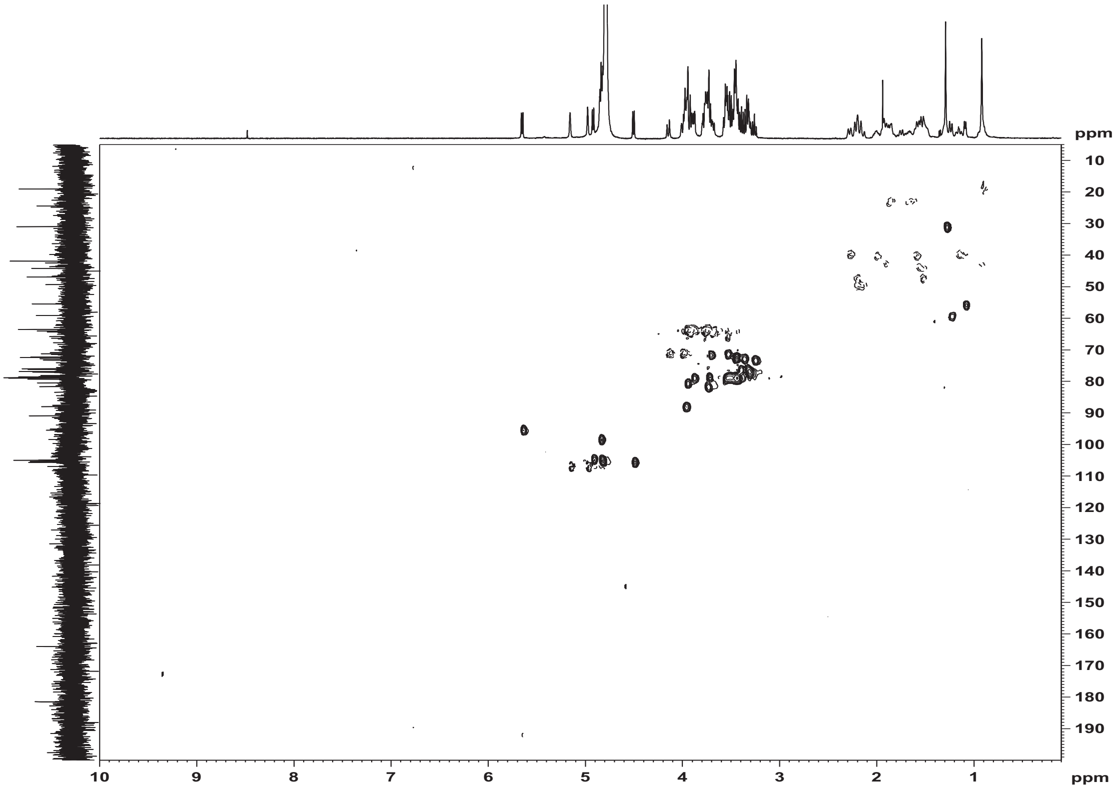

Figure S1. *Cont.*

E

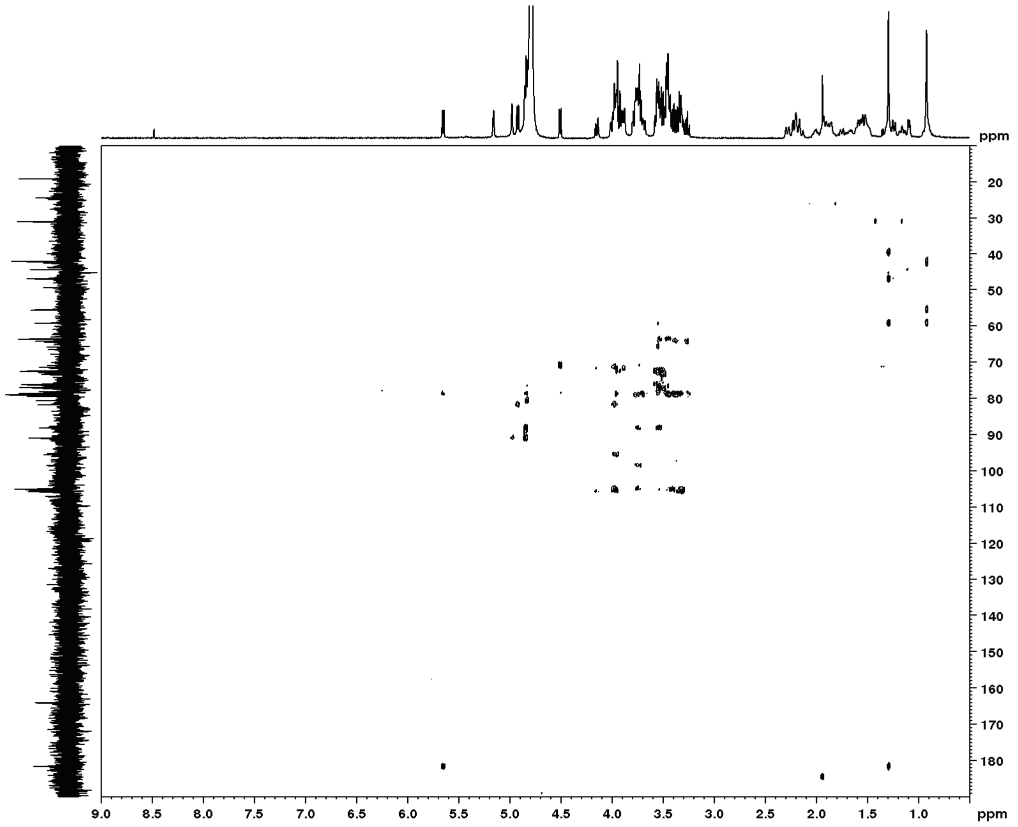

F

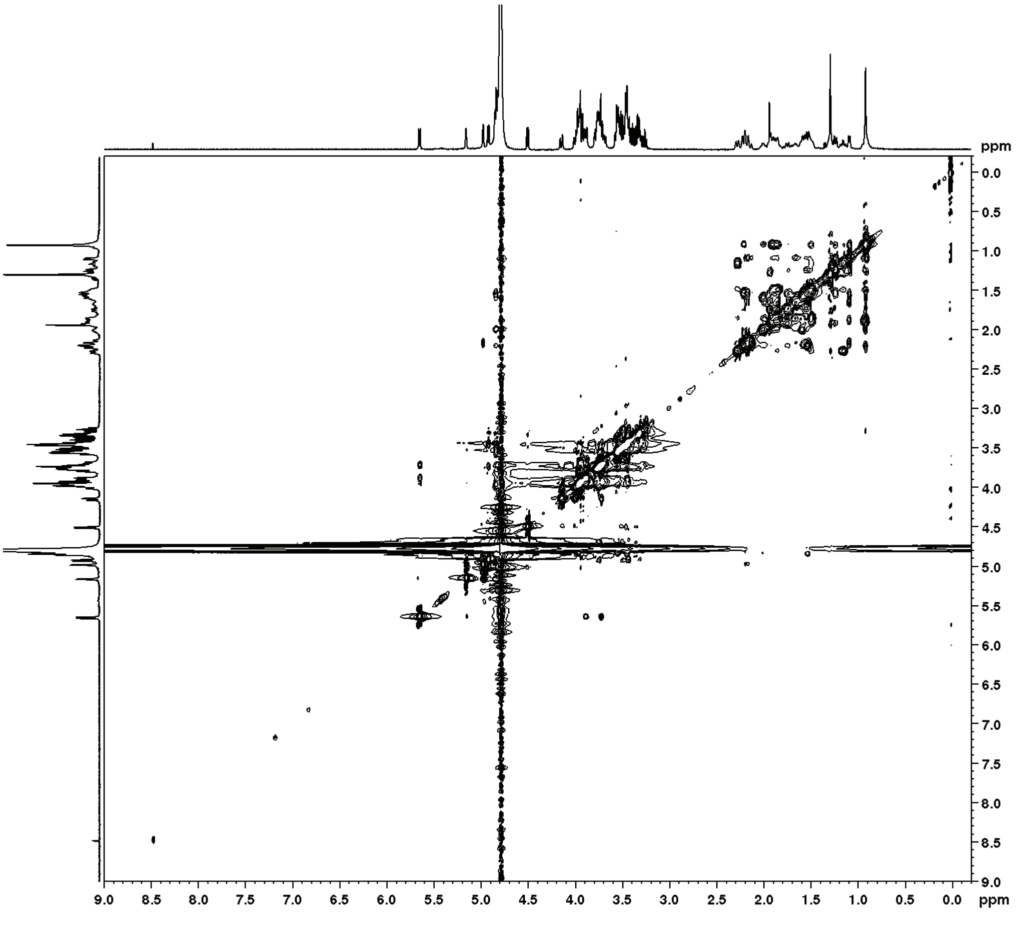

Figure S2. 1D and 2D NMR spectra of Rebaudioside M (**1**); (**A**) ^{1}H-NMR spectrum of **1**; (**B**) ^{13}C-NMR spectrum of **1**; (**C**) ^{1}H-^{1}H COSY spectrum of **1**; (**D**) ^{1}H-^{13}C HSQC-DEPT spectrum of **1**; (**E**) ^{1}H-^{13}C HMBC spectrum of **1**.

A

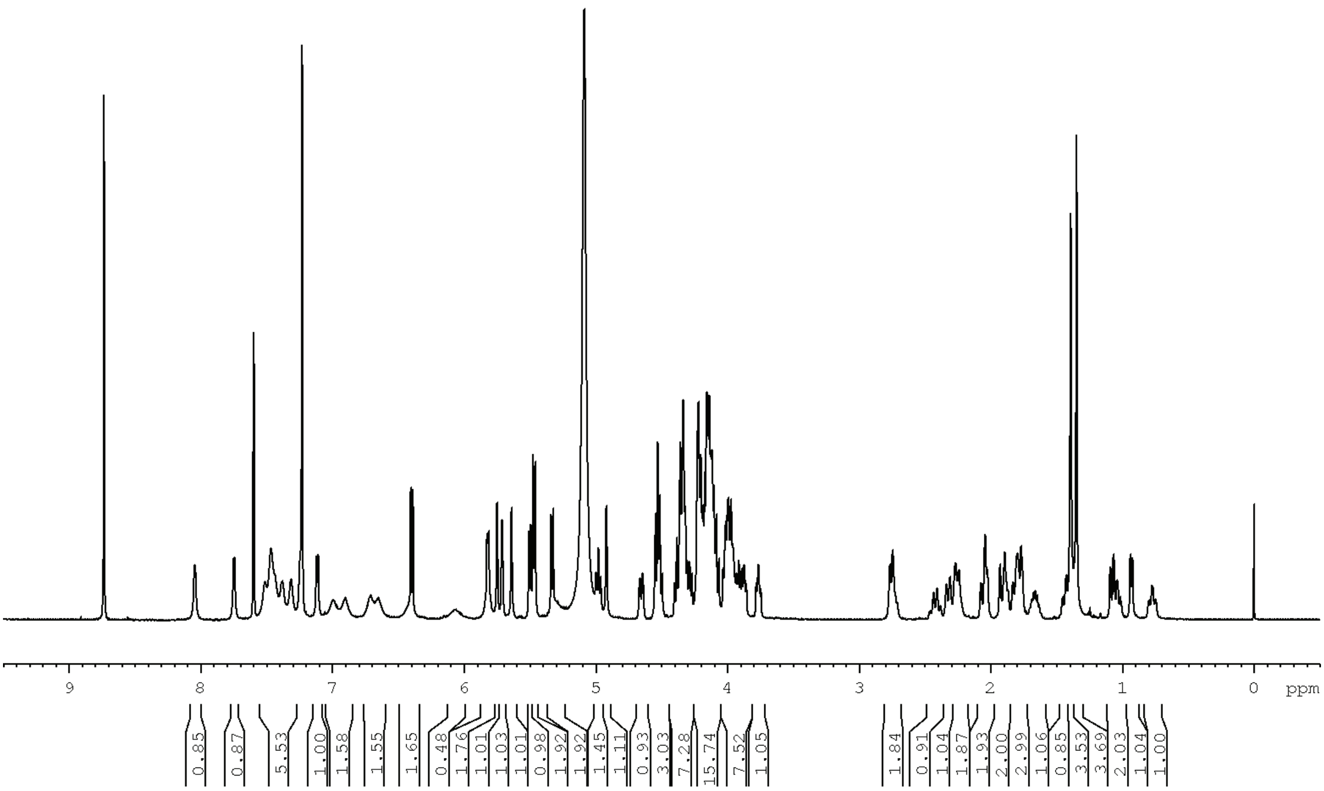

B

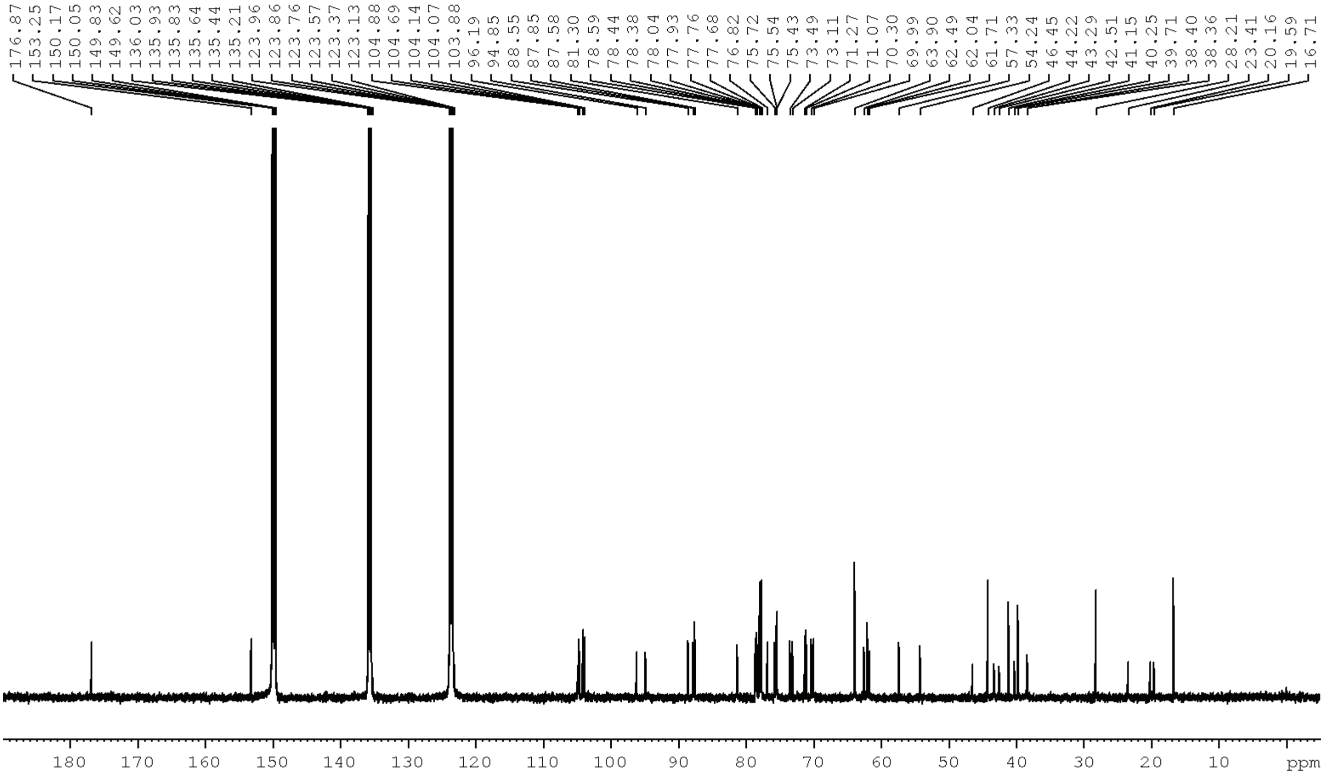

Figure S2. *Cont.*

C

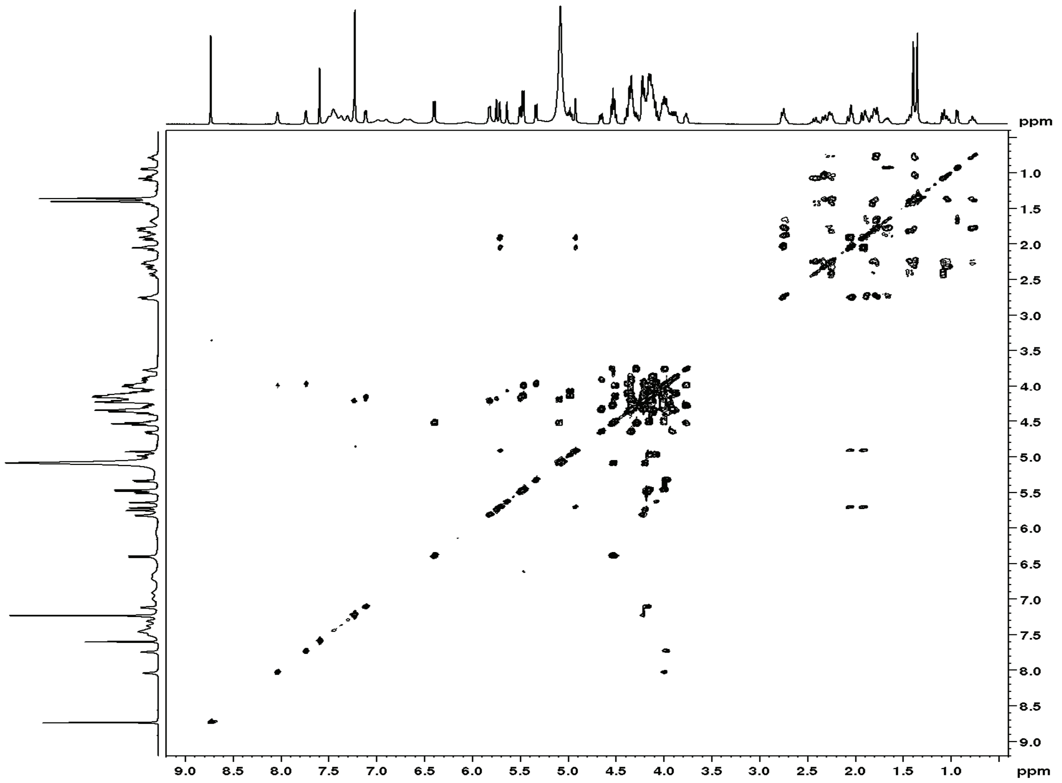

D

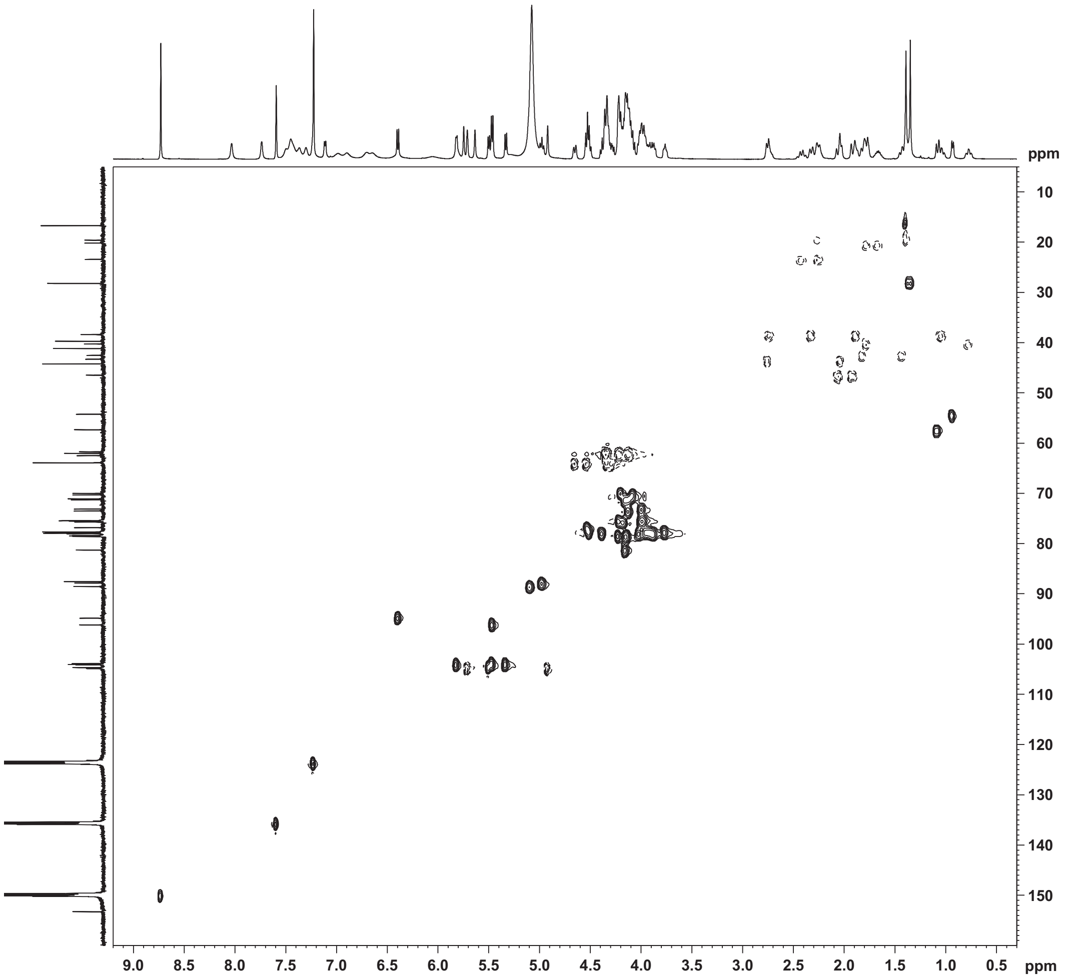

Figure S2. *Cont.*

E

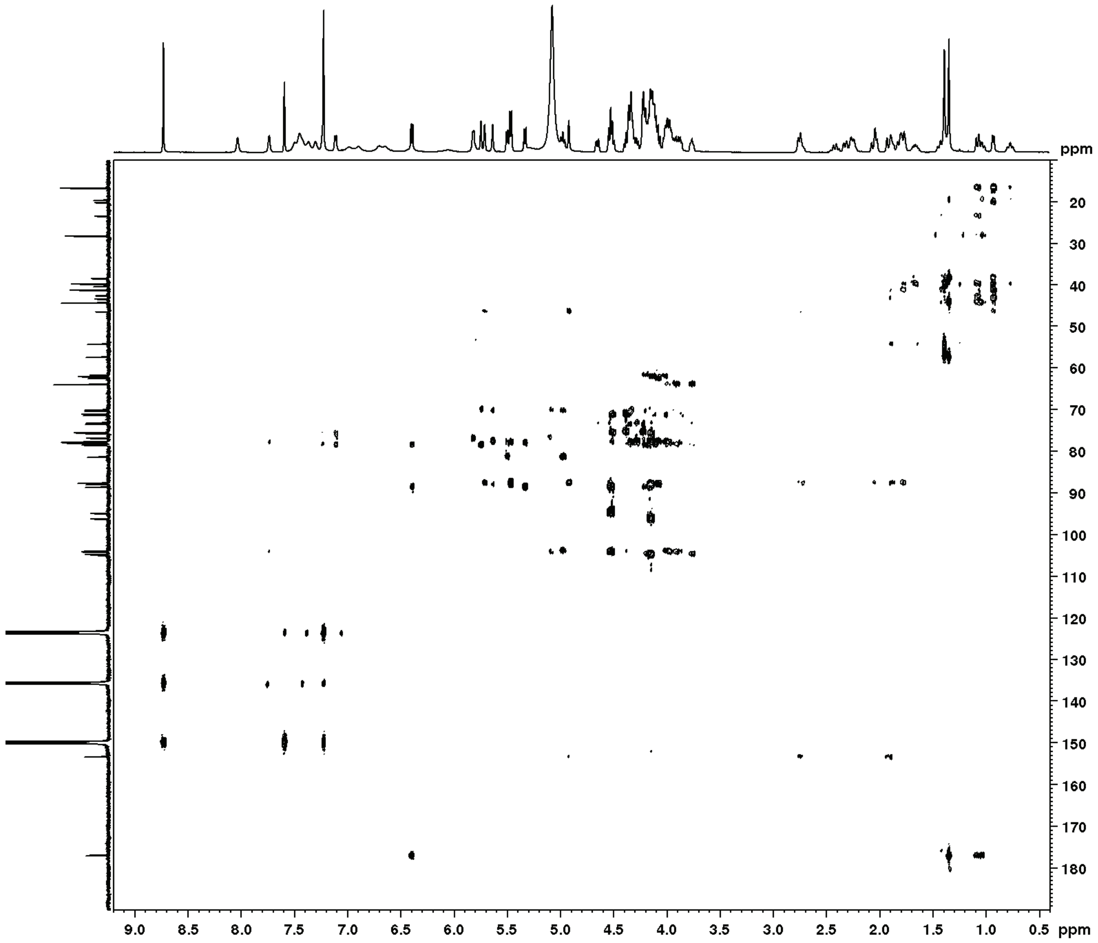

Figure S3. ^{1}H- and ^{13}C-NMR spectra of Rebaudioside D (**3**). (**A**) ^{1}H-NMR spectrum of **3**; (**B**) ^{13}C-NMR spectrum of **3**.

A

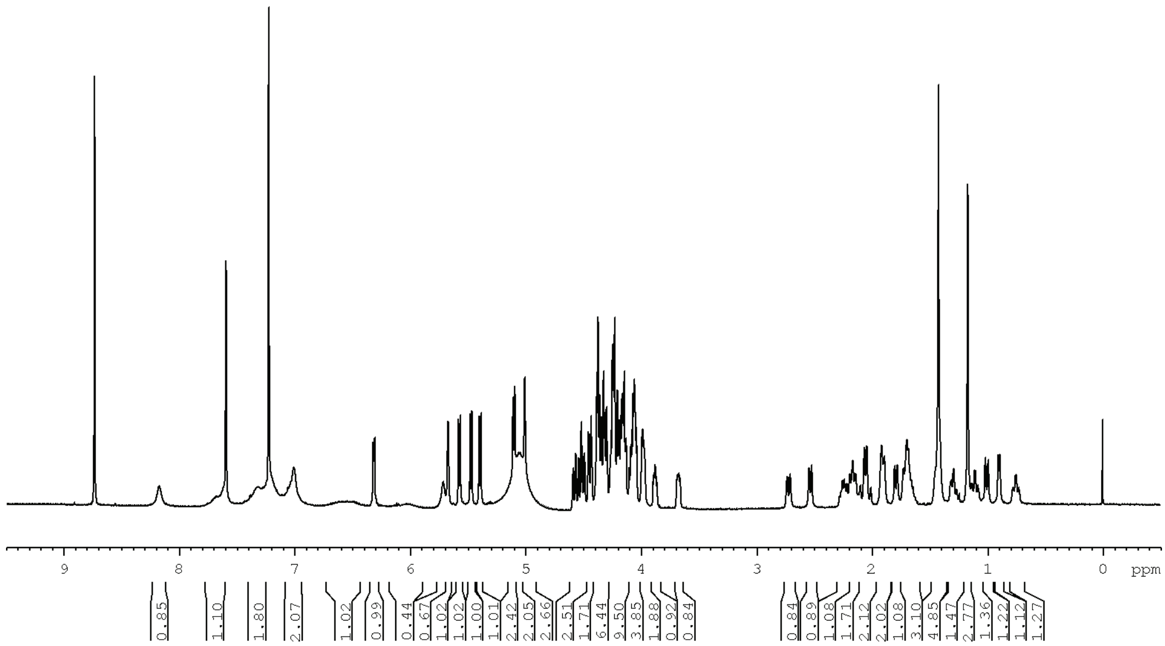

Figure S3. *Cont.*

B

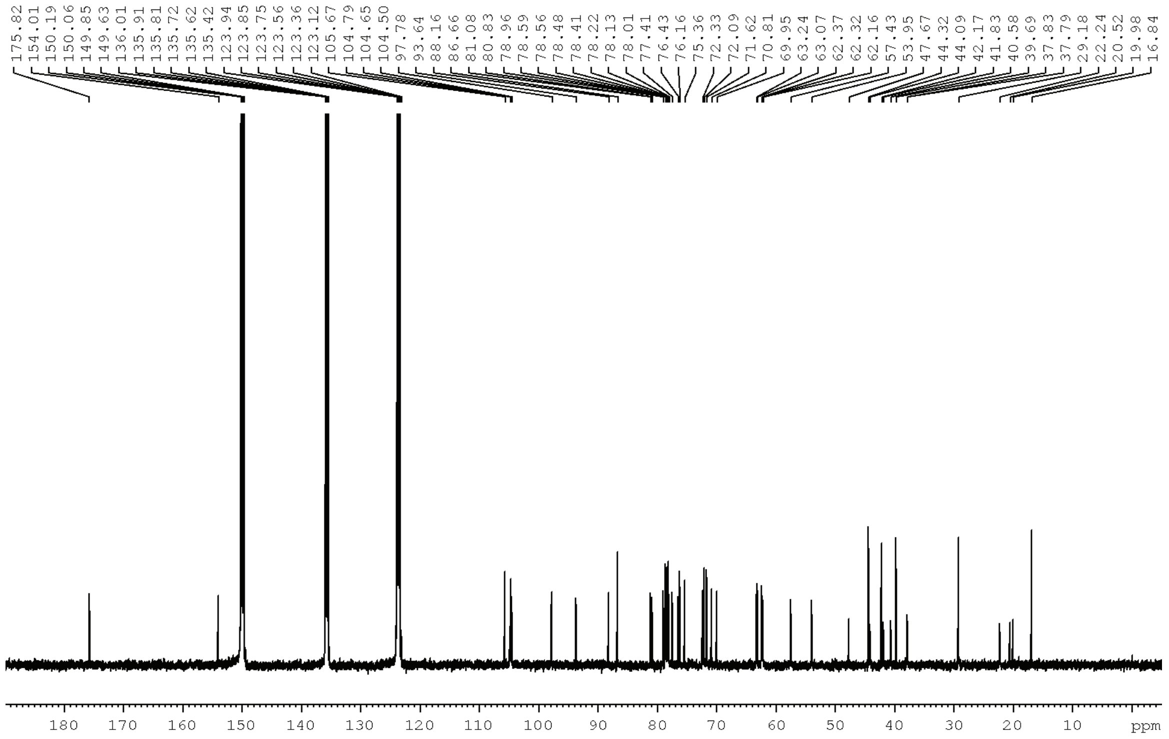

Figure S4. ^{13}C, HSQC-DEPT, and HMBC NMR spectra of 82% Rebaudioside M (**1**) + 18% Rebaudioside D (**3**). (**A**) ^{13}C-NMR spectrum of 82% Rebaudioside M (**1**) + 18% Rebaudioside D (**3**); (**B**) Expansion of ^{13}C-NMR spectrum of 82% Rebaudioside M (**1**) + 18% Rebaudioside D (**3**) (13–60 ppm); (**C**) Expansion of ^{13}C-NMR spectrum of 82% Rebaudioside M (**1**) + 18% Rebaudioside D (**3**) (85–108 ppm); (**D**) Expansion of ^{13}C-NMR spectrum of 82% Rebaudioside M (**1**) + 18% Rebaudioside D (**3**) (152–180 ppm); (**E**) HSQC-DEPT NMR spectrum of 82% Rebaudioside M (**1**) + 18% Rebaudioside D (**3**); (**F**) HMBC NMR spectrum of 82% Rebaudioside M (**1**) + 18% Rebaudioside D (**3**).

A

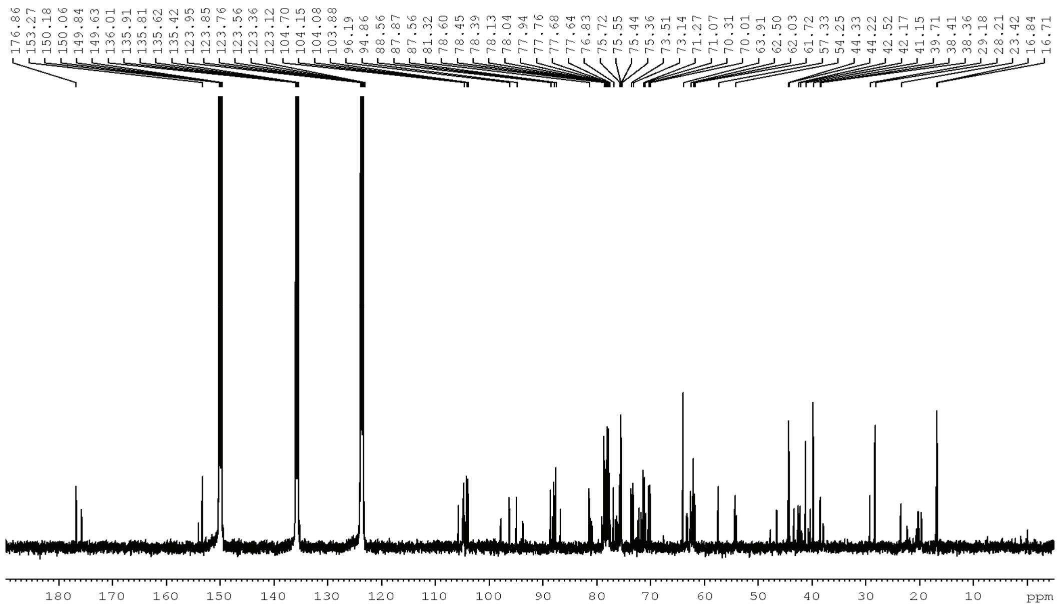

Figure S4. *Cont.*

B

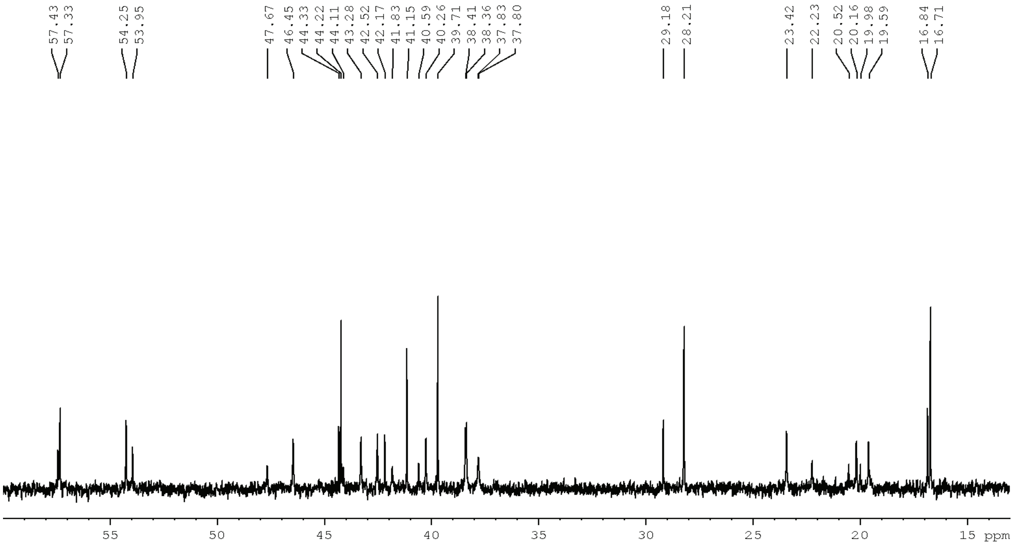

C

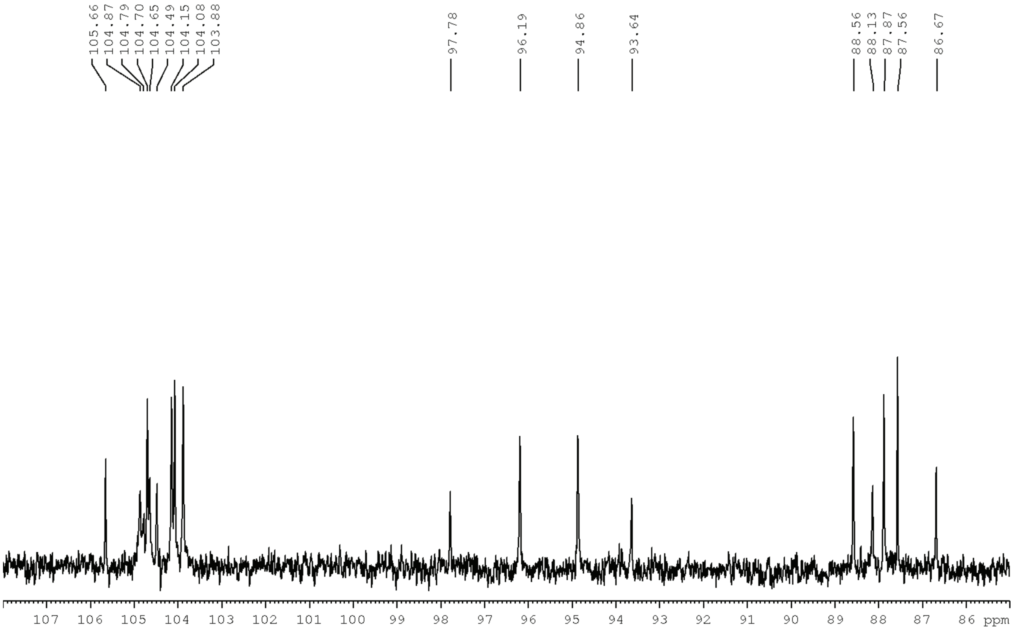

D

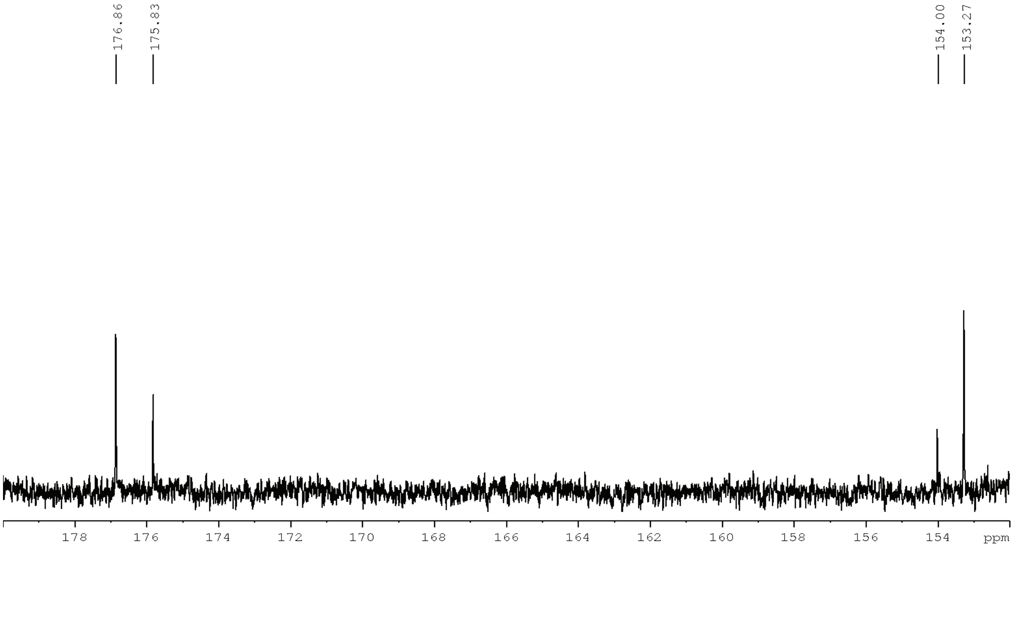

Figure S4. *Cont.*

E

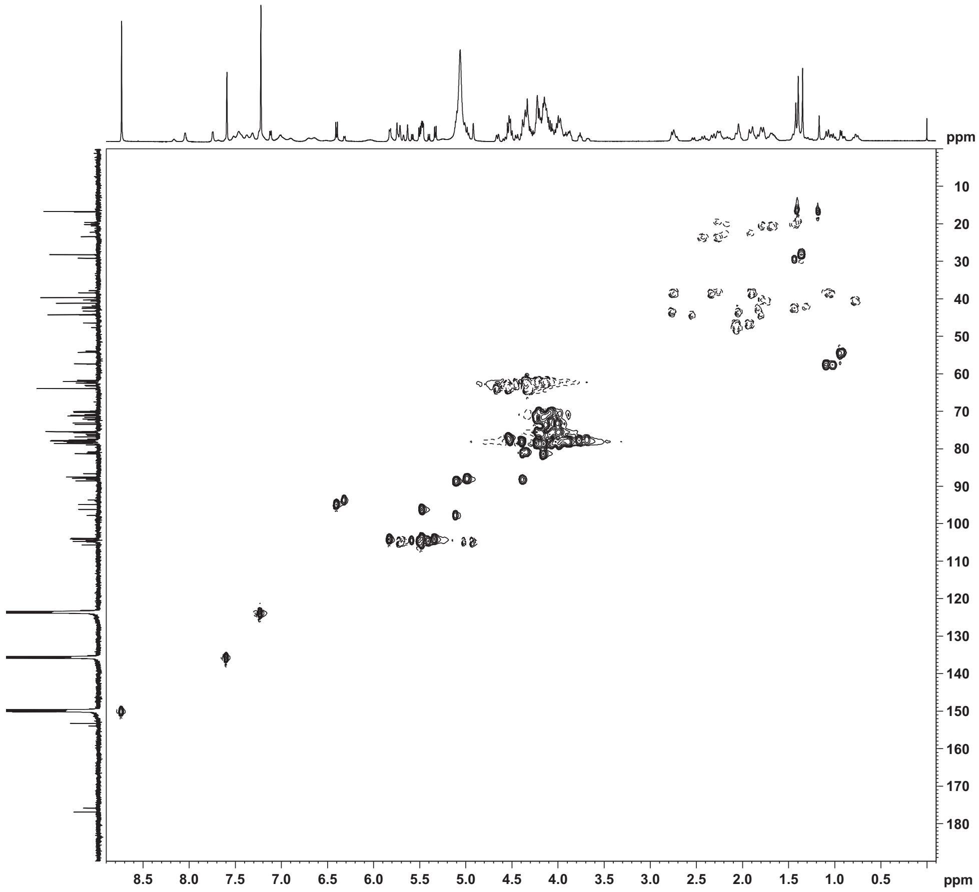

F

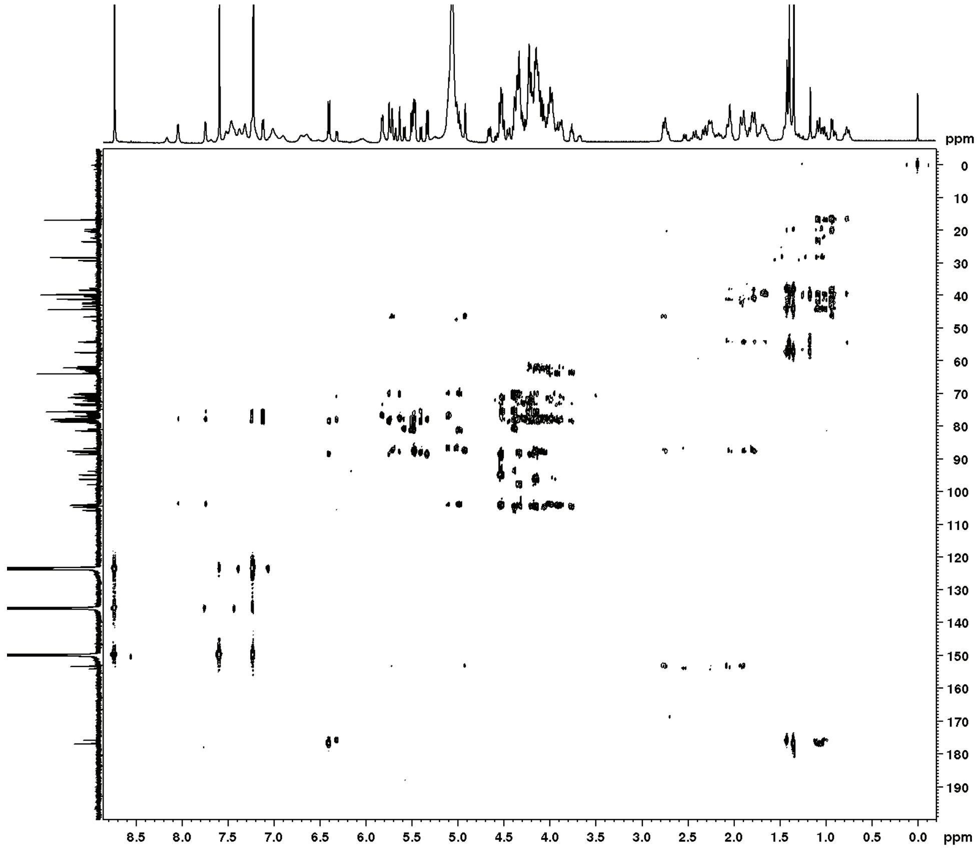

Figure S5. ^{13}C and HSQC-DEPT NMR spectra of 82% Rebaudioside D (**3**) + 18% Rebaudioside M (**1**). (**A**) ^{13}C-NMR spectra of 82% Rebaudioside D (**3**) + 18% Rebaudioside M (**1**); (**B**) Expansion of ^{13}C-NMR spectra of 82% Rebaudioside D (**3**) + 18% Rebaudioside M (**1**) (13–60 ppm); (**C**) Expansion of ^{13}C-NMR spectra of 82% Rebaudioside D (**3**) + 18% Rebaudioside M (**1**) (85–108 ppm); (**E**) HSQC-DEPT NMR spectra of 82% Rebaudioside D (**3**) + 18% Rebaudioside M (**1**).

A

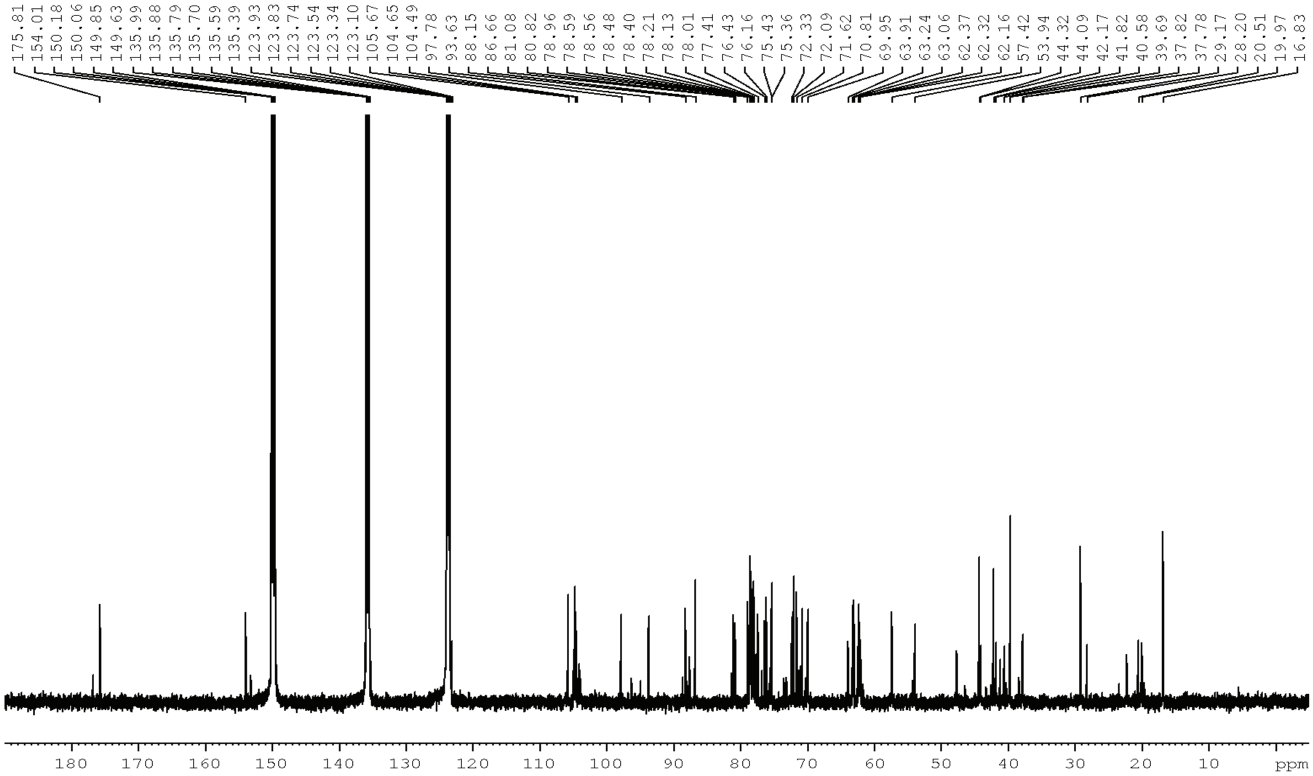

B

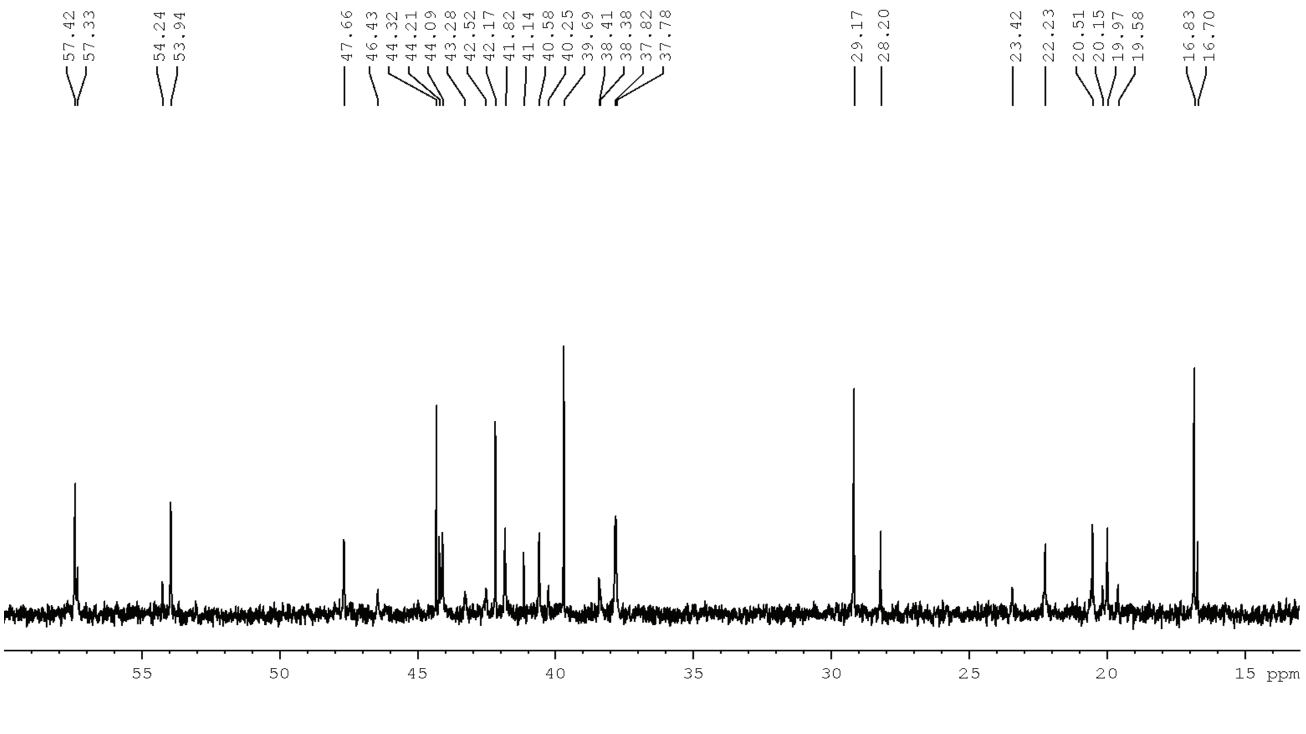

Figure S5. *Cont.*

C

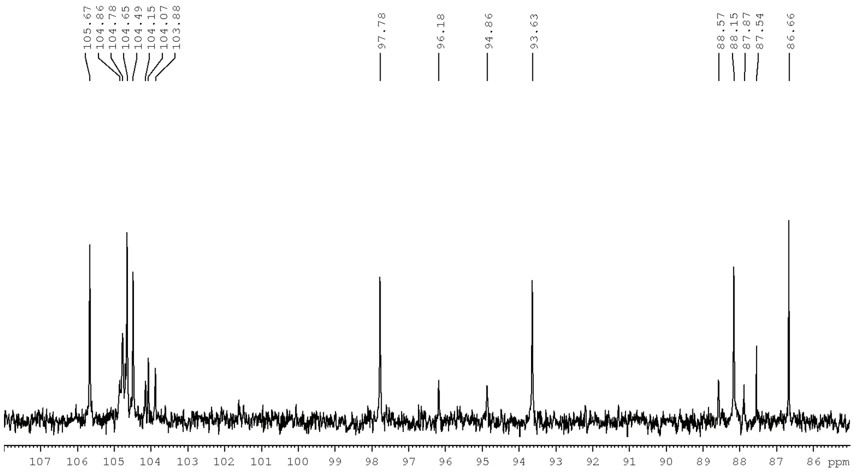

D

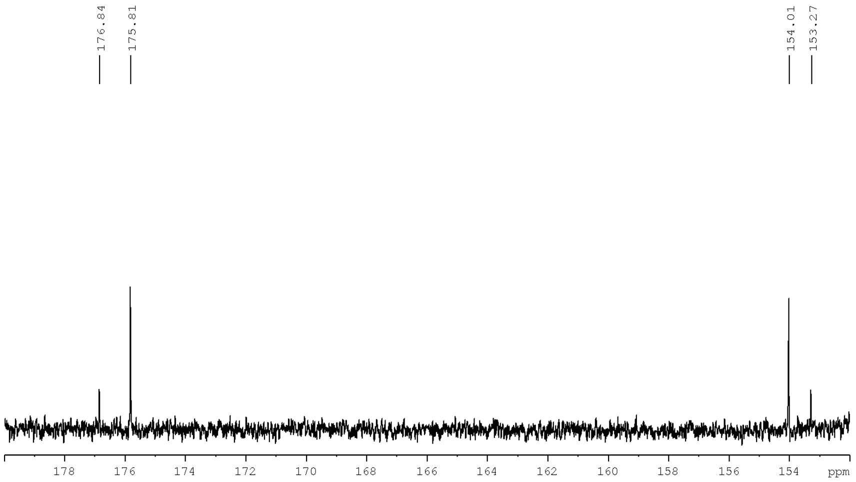

E

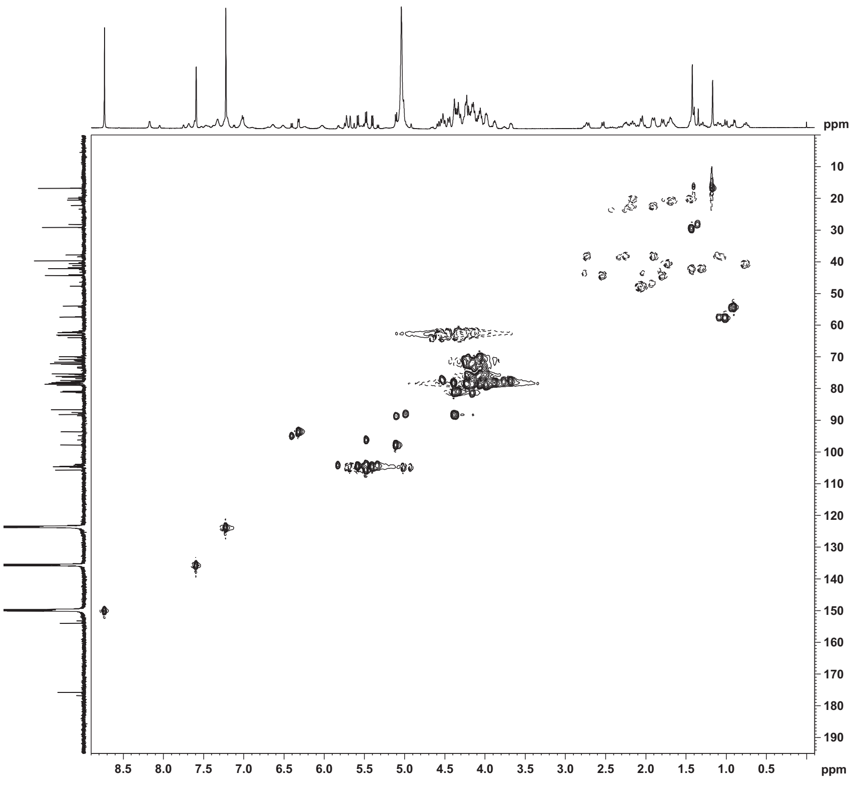

Figure S6. ^{13}C-NMR spectra of 80% Rebaudioside M (**1**); (**A**) ^{13}C-NMR spectra of 80% Rebaudioside M (**1**); (**B**) Expansion of ^{13}C-NMR spectra of 80% Rebaudioside M (**1**) (13–60 ppm); (**C**) Expansion of ^{13}C NMR spectra of 80% Rebaudioside M (**1**) (85–108 ppm); (**D**) Expansion of ^{13}C-NMR spectra of 80% Rebaudioside M (**1**) (152–180 ppm).

A

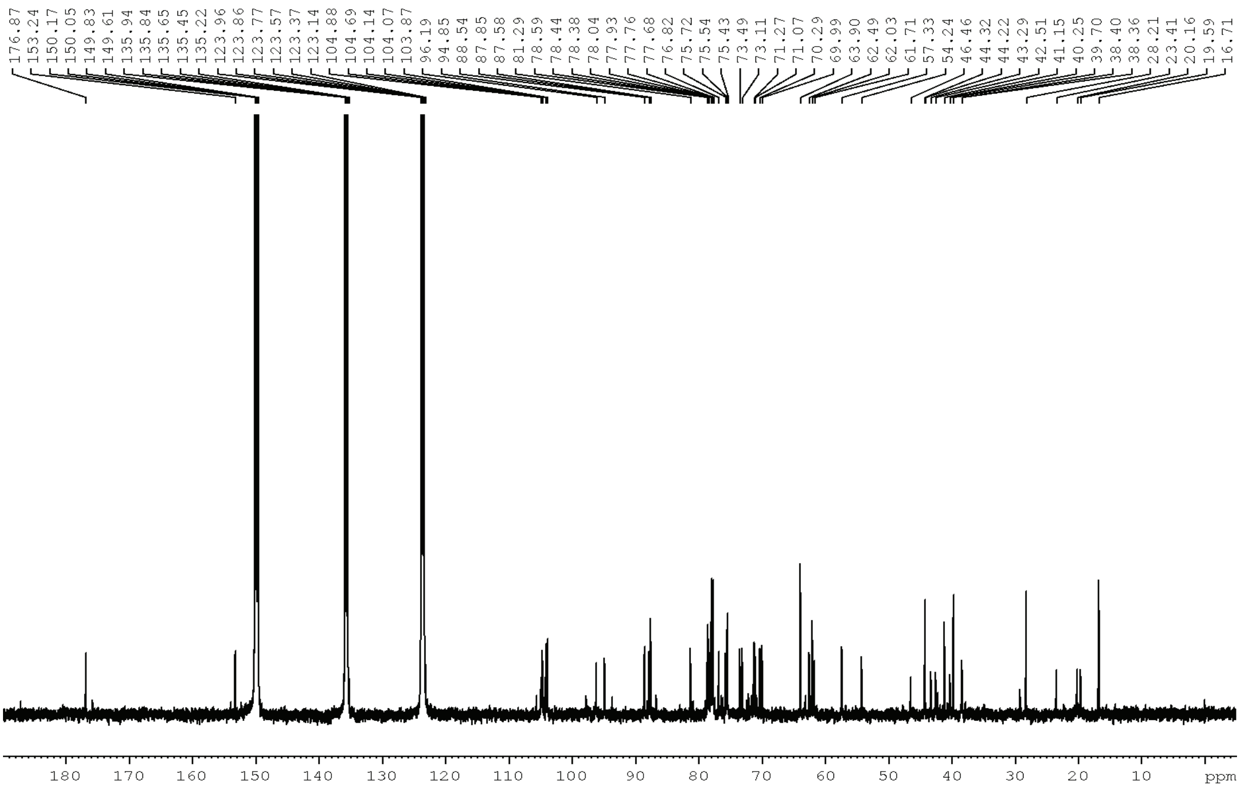

B

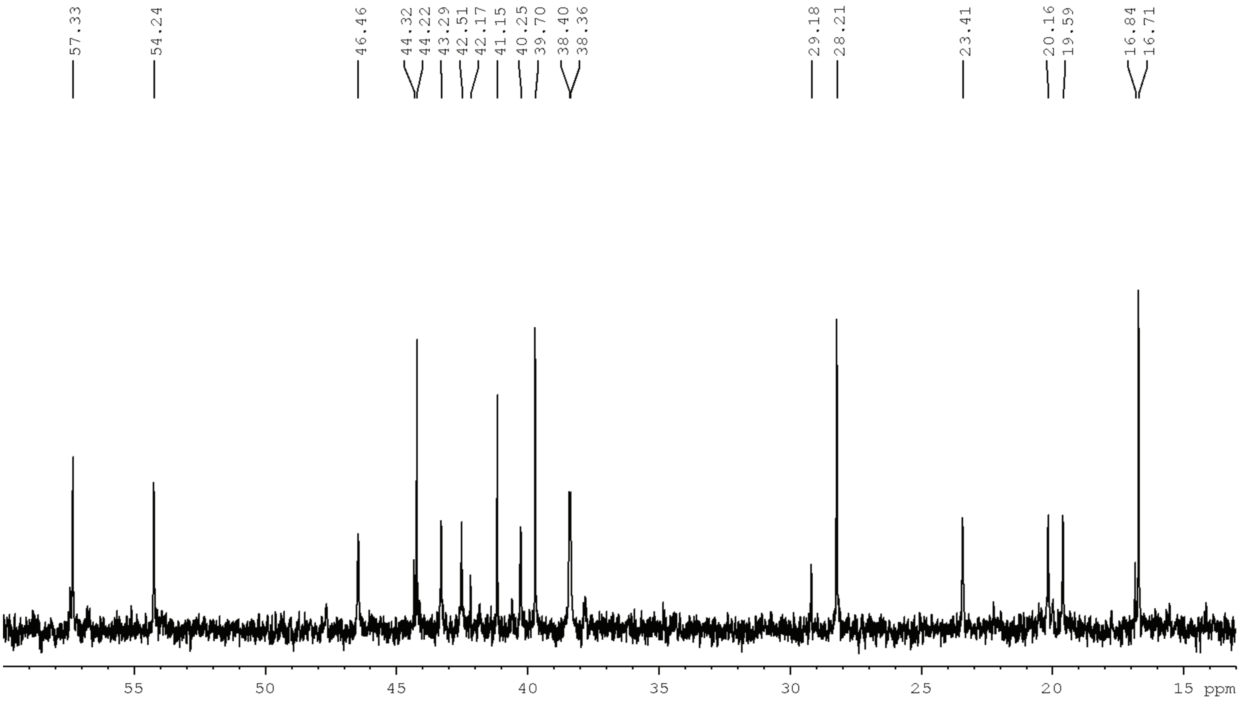

Figure S6. *Cont*

C

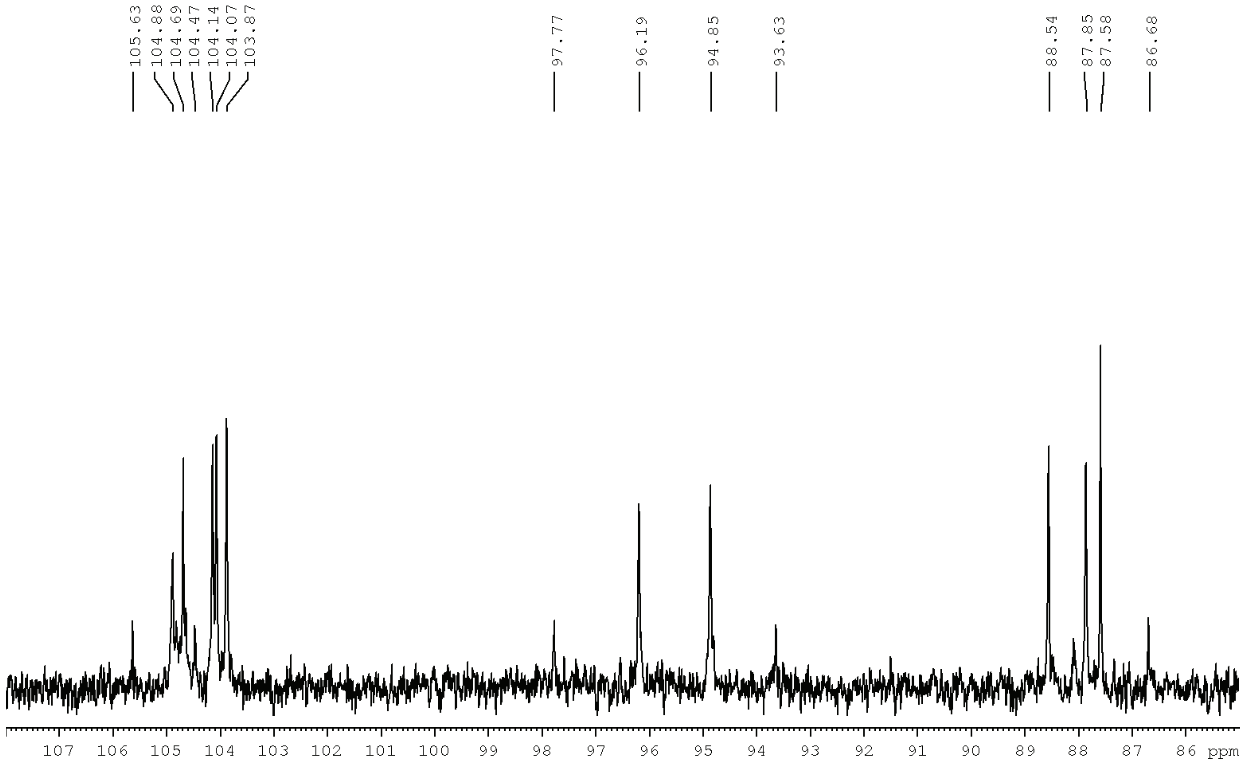

D

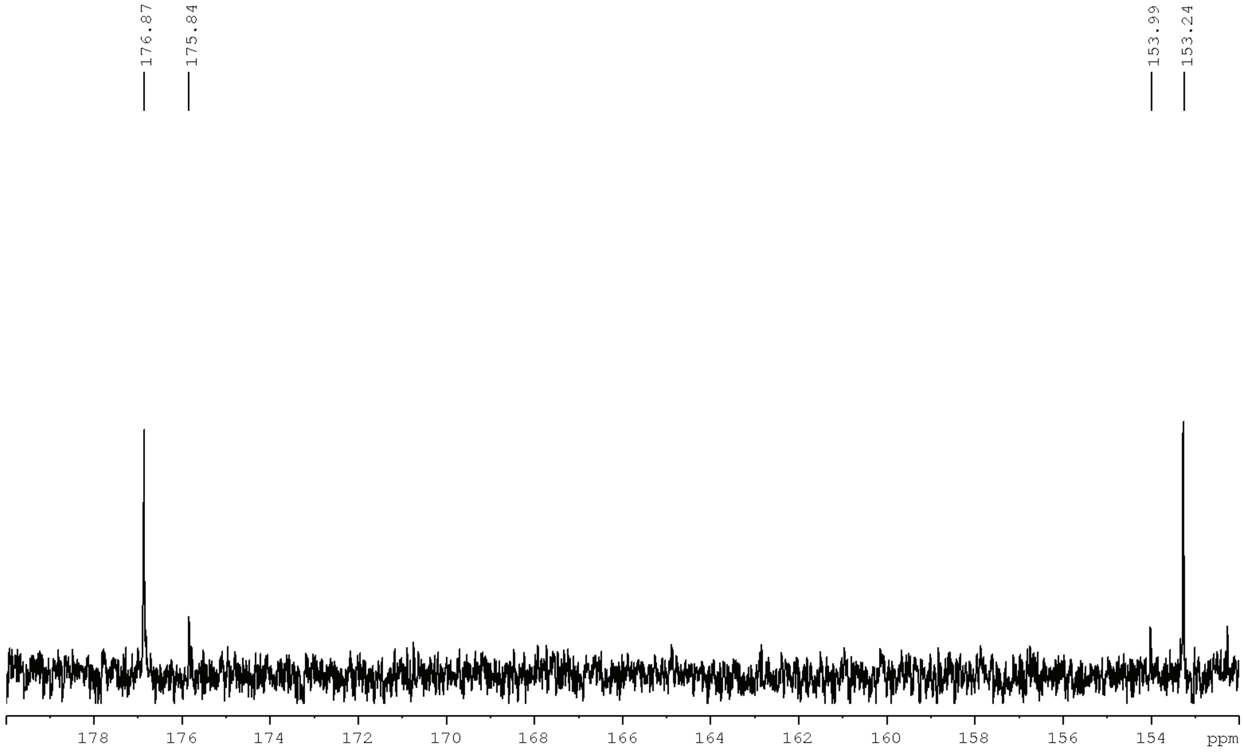

© 2014 by the authors; licensee MDPI, Basel, Switzerland. This article is an open access article distributed under the terms and conditions of the Creative Commons Attribution license (http://creativecommons.org/licenses/by/3.0/).
